# Spatiotemporal regulation by downstream genes of *Prok2* in the olfactory system: from development to function

**DOI:** 10.3389/fcell.2025.1550845

**Published:** 2025-07-22

**Authors:** Bo-Ra Kim, Min-Seok Rha, Hyung-Ju Cho, Joo-Heon Yoon, Chang-Hoon Kim

**Affiliations:** ^1^The Airway Mucus Institute, Yonsei University College of Medicine Seoul, Seoul, Republic of Korea; ^2^Department of Otorhinolaryngology, Yonsei University College of Medicine, Seoul, Republic of Korea; ^3^Korea Mouse Sensory Phenotyping Center, Yonsei University College of Medicine, Seoul, Republic of Korea; ^4^Human Microbiome Center, Yonsei University College of Medicine, Seoul, Republic of Korea

**Keywords:** prokineticin 2 gene, kallman syndrome, olfactory dysfunction, olfactory sensory neuron, embryonic development, spatiotemporal regulation, Transcriptomic (RNA-Seq), intermediate filament (IF)

## Abstract

**Introduction:**

Olfaction is important for the quality of life; however, in Kallmann syndrome (KS), defective development results in olfactory dysfunction. Notably, the mechanism underlying olfactory development, especially in the olfactory epithelium (OE), which detects olfactory signals, remains unclear. Mutations in PROK2, which encodes prokineticin-2, have been identified in approximately 9% of the KS patients with olfactory defects.

**Methods:**

We examined olfactory function and analyzed the causes of olfactory dysfunction based on spatiotemporal development and gene expression changes in *Prok2* knockout (KO) model mice with KS.

**Results:**

The ability of the OE to detect olfactory signals was diminished in adult *Prok2* KO mice. Maturation of olfactory sensory neurons (OSNs) in the OE and formation of glomeruli in the olfactory bulb (OB) in adult *Prok2* KO mice were disrupted, thus causing olfactory dysfunction. Furthermore, molecular analysis of *Prok2* KO mice during embryonic development revealed abnormal development of OB layers and diminished differentiation to mature OSNs in the OE at the later stage, which caused defects in the entire olfactory system. Remarkably, downstream signaling genes of *Prok2*, including intermediate filament genes and genes expressed in the putative OB, were found to mediate olfactory system organization.

**Discussion:**

Overall, these findings reveal the role of *Prok2* in olfactory system organization and elucidate how olfactory development defects translate to olfactory function.

## 1 Introduction

Mammals depend on their sense of smell to obtain essential nutrients, recognize danger, recall memories, and derive pleasure (Ghibaudo et al., 2024; Herz and Engen, 1996; Kadohisa, 2013; Lee et al., 2024; Parvin et al., 2024; Santos et al., 2004). Therefore, olfactory dysfunction is detrimental to health and survival as it can result in nutritional deficiencies, consumption of spoiled food, exposure to hazardous chemicals or fire, and inability to maintain positive emotions, which can lead to depression, anxiety, and social isolation.

Olfactory dysfunction may be a result of brain disorders, including brain tumors or traumatic brain injury (Li et al., 2023; Proskynitopoulos et al., 2016), structural abnormalities in the nasal cavity caused by inflammation (Gudis and Soler, 2016), neuronal degeneration caused by pathogenic infection or neurodegenerative diseases (Murphy, 2019; Tsukahara et al., 2023), and abnormal development in olfactory system due to genetic variations (Dodé and Rondard, 2013; Topan et al., 2021). In particular, developmental diseases caused by abnormal development lead to congenital olfactory disorders. Therefore, the genes that contribute to the organization of the olfactory system can be identified by studying the developmental disorders with olfactory dysfunction.

Kallmann syndrome (KS) is a developmental disease with hypogonadotropic hypogonadism and congenital olfactory dysfunction (Dodé and Rondard, 2013; Forni and Wray, 2015; Rugarli, 1999). KS mainly occurs as a result of the failure of gonadotropin-releasing hormone (GnRH) neurons to reach the brain in the early developmental stage, resulting in impaired formation of the reproductive axis and construction of olfactory bulb (OB) (Barraud et al., 2013; Schwanzel-Fukuda et al., 1994; Taroc et al., 2020a; b; Taroc et al., 2017; Watanabe et al., 2009). In addition, the mutation of *anosomin-1* (*Kal-1*), *Prok2*, *Prokr2*, *Fgf8*, *Fgfr1*, *Gli3*, *Isl1*, *Sox10*, *Fezf1*, and *Sema3a* leads to complications in the migration of GnRH neurons, and most of these genes regulate the formation of the nasal forebrain junction (NFJ) (Dodé and Rondard, 2013; Taroc et al., 2020a; b; Watanabe et al., 2009). Among the mutant genes, *Prok2* knockout (KO) exhibits the core features of KS, including OB hypoplasia, defective GnRH migration, and reproductive defects, with high similarity to patients with KS and higher survival rate than that of other KS mutant mouse models. Therefore, *Prok2* KO can be considered a representative mouse model of KS.

Prokineticin 2 (Prok2) is a secretory protein that interacts with prokineticin receptor 2 (Prokr2) (Dodé and Rondard, 2013). Moreover, Prok2/Prokr2 signaling influences GnRH migration from the vomeronasal organ (VNO) to the OB and the migration of neuronal stem cells from the subventricular zone to the OB (Ng et al., 2005; Pitteloud et al., 2007; Wen et al., 2019). However, the defects present in the olfactory epithelium (OE), which detects olfactory stimuli, and the molecule that modulates the formation of the olfactory system during olfactory development remain unexplored. Therefore, to elucidate the causal mechanism of olfactory disorders, we studied the changes in the olfactory structure during embryonic development and identified the genes regulated by *Prok2* using *Prok2* KO mice as a KS disease model.

## 2 Materials and methods

### 2.1 Mice

The *Prok2* KO mice used in this study were purchased from UC Davis (Davis, CA, United States). Homozygous KO mice were distinguished from littermate wild-type (WT) mice via genotyping with the sequences WT-F, WT-R, KO-F, and KO-R described in Supplementary Table S1. Most homozygous KO mice are mostly produced via *in vitro* fertilization involving the sperms and eggs of hemizygote KO mice because they cannot be obtained easily by natural mating. E11.5, 13.5, 14.5, or 18.5 embryos were acquired on day 11, 13, 14, or 18, respectively, after detecting the plug in female mice. Mice have been handled and maintained in strict accordance with the guidelines for the Care and Use of Laboratory Animals of Yonsei University College of Medicine. The research was performed under the Yonsei Medical Center Animal Research Guidelines (IACUC No. 2023-0184), which adhere to the standards articulated in the Association for Assessment and Accreditation of Laboratory Animal Care International (AAALAC) guidelines.

### 2.2 Manganese-enhanced magnetic resonance imaging (MEMRI)

MEMRI was performed on mice aged 16 weeks based on a previous study (Pautler and Koretsky, 2002). After the mice were initially anesthetized with 3% isoflurane, 20 μL of 10 mM MgCl_2_ was administered nasally. The anesthetized mice were maintained on 1.5%–2% isoflurane and imaged at 20 min after treatment with MgCl_2_ using a 9.4T Advanced imaging spectrometer (Bruker, Billerica, MA, United States) with a 4 cm microimaging gradient insert. The imaging parameters were as follows: TR = 2000.0 ms, TE = 22.0 ms, FOV = 1.28 × 1.28 × 0.64 cm, and matrix dimensions 128 × 128 × 64. The nominal resolution of each plane was 130 μm.

### 2.3 Olfactory behavior test

**FIGURE 1:**
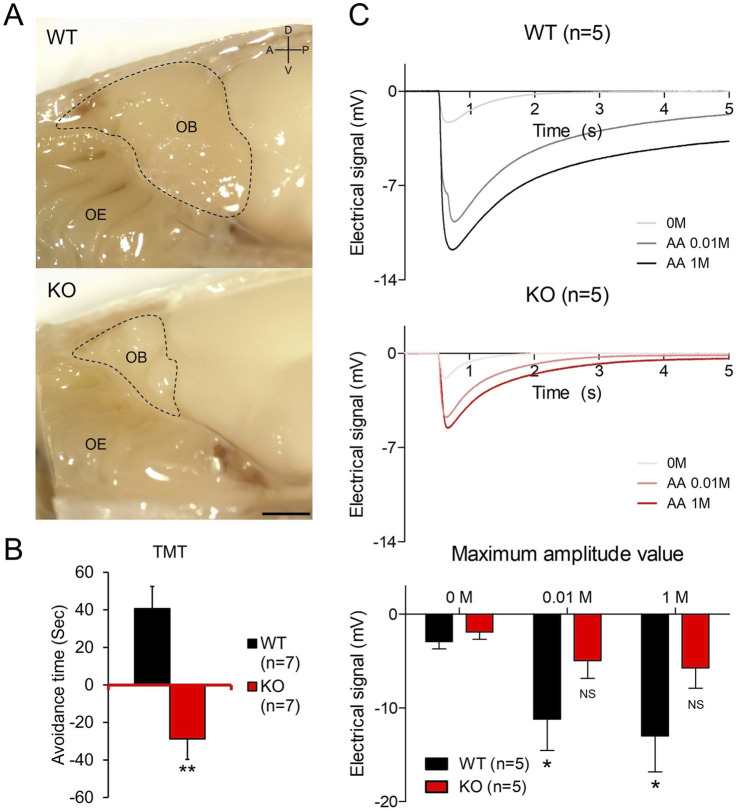
Olfactory bulb hypoplasia and olfactory dysfunction in *Prok2* KO **(A)** A sagittal view of the olfactory bulb (OB) structure in WT and *Prok2* KO mice. Each dashed line represents the OB size. D, dorsal; V, ventral; A, anterior; P, posterior. Scale bar, 1 cm. **(B)** Graph of the avoidance test. Differences in avoidance time were considered significant at p = 0.0010 in WT (n = 7) and *Prok2* KO mice (n = 7). Data are presented as mean ± SD. **(C)** Graphs of time-dependent electrical signals in WT (n = 5) and *Prok2* KO mice (n = 5) and the graph of dose-dependent electrical signals in WT (n = 5) and *Prok2* KO mice (n = 5). In WT mice, the difference between the electrical signals at 0 M and at 0.01 M was significant at p = 0.0417, and that between electrical signals at 0 M and at 1 M was significant at p = 0.0327. In *Prok2* KO mice, the difference between the electrical signals at 0 M and at 0.01 M was significant at p = 0.3505, and that between the electrical signals at 0 M and at 1 M was significant at p = 0.1301. Data are presented as mean ± SD. In all panels, n indicates biologically independent repeats. P values were determined using two-tailed unpaired Student’s t-test to compare two groups; *, p < 0.05; **, p < 0.01; ns, non-significant.

Olfactory behavior test was performed in this study based on the avoidance test described in previous papers (Cho et al., 2018; Kobayakawa et al., 2007). Mice were tracked and the time spent by each mouse in each zone were automatically measured by a smart video tracking system (Smart 3.0; SHarvard Apparatus^©^, Holliston, MA, United States). All procedures were conducted between 8 a.m. and 12 p.m., which was close to the dark cycle of the mice. Trimethylthiazoline (TMT, purchased from BioSRQ) diluted in water was used as the odorant. In the habituation step, mice were placed in the test cage for 10 min and transferred back to the home cage (i.e., the cage in which the mouse resides) for 5 min before the test. The test cage was divided into equal halves using a curtain. To perform the test, mice were laid in the zone containing 20 μL of 10% TMT or water in the test cage for 3 min. The avoidance time plotted in the graph was calculated by subtracting each time spent avoiding TMT from the sum of the time spent avoiding water. The red bar in the graph (Figure 1B) indicates the avoidance time in relation to water. The experimental results are presented as mean ± SD. Statistical analysis was performed using a t-test in GraphPad Prism 5.

### 2.4 Electro-olfactogram (EOG)

EOG was obtained as described before (Cygnar et al., 2010). Olfactory tissue samples were prepared from the hemi-sectioned head of mice, and the OE in these samples were visualized in the sagittal view immediately after the mice were decapitated. Tissues were maintained in *ex vivo* conditions using humid and warm air in order to detect the electrical signal analogous to the live status. Next, 0.01 M and 1 M amyl acetate (Sigma, #W504009) were used as the odorant. A 5–10-μm pore size pipette was hardened using 0.05% agarose and filled with Ringer’s solution (135 mM NaCl, 5 mM KCl, 1 mM CaCl_2_, 1.5 mM MgCl_2_, and 10 mM HEPES, pH 7.4, sterilized via filtration). The point of the quarter region in the secondary turbinate of the OE was measured. The pipette was placed at the measured region after confirming that the voltage changed when the tissue came in contact with the pipette. Then, the voltage was recorded continuously. After 0.95 s, the OE were exposed to the stimuli vehicle, 0.01 M amyl acetate, or 1 M amyl acetate in that order. Recording was continued up to 10 s. The graphs were plotted, and statistical analysis was performed using GraphPad Prism 5. The maximum amplitude value in the graph (Figure 1C) was presented as the maximum of the continuous values and as mean ± SD.

### 2.5 Immunostaining

Slides were prepared by paraffin sectioning or cryosectioning. In the case of paraffin-sectioned slides, the following procedure was performed before blotting them with antibodies: deparaffinization, antigen retrieval with the solution (#IW-1100; IHC World, Ellicott City, MD, United States) in a steaming bowl (IW-1102; IHC World) for 40 min, incubation with hydrogen peroxide (#3059; Duksan, Korea) for 10 min to quench endogenous peroxidase, and washing with tris-buffered saline (TBS) thrice for 5 min each. In the case of cryosectioned slides, antigen retrieval for 10 min and washing with TBS was performed sequentially. After blocking with 5% bovine serum albumin for 1 h, slides were blotted with primary antibodies for 1 h at room temperature or overnight at 4°C. The primary antibodies used are described in Supplementary Table S2. The slides were washed and then blotted with fluorescent secondary antibodies (Thermo Fisher Scientific, Waltham, MA, United States) for 30 min at room temperature. The slides were washed again and mounted with the medium containing DAPI (#F6057; Sigma-Aldrich, St. Louis, MO, United States).

All experiments were conducted using at least three biological replicates from independent experiments.

### 2.6 Transmission electron microscopy (TEM)

Olfactory tissues were fixed with Karnovsky’s fixative (2% glutaraldehyde and 2% paraformaldehyde (PFA) in 0.1 M phosphate buffer, pH 7.4) for 1 day. Decalcification was performed using 10% EDTA for 2 weeks. After washing with 0.1 M phosphate buffer, tissues were fixed with 1% OsO_4_ in 0.1 M phosphate buffer for 2 h, dehydrated, and infiltrated with propylene oxide for 10 min. For making blocks, tissues were embedded with a Poly/Bed 812 kit (Polysciences, Warrington, PA, United States) and polymerized in an electron microscope oven (TD-700; DOSAKA, Japan) at 65°C for 12 h. Blocks were cut into 80 nm pieces and placed on copper grids. Then, the sections were doubly stained with 3% uranyl acetate for 30 min and 3% lead citrate for 7 min. Sections were observed using a microscope (JEM-1011; JEOL, Tokyo, Japan) equipped with a Megaview III CCD camera (Soft Imaging System, Germany) at an acceleration voltage of 80 kV. The graphs were drawn, and statistical analysis was performed using a t-test with GraphPad Prism 5.

### 2.7 *In situ* hybridization (ISH)

To create probes, the sequences of each target were amplified by PCR using IP pro-Taq (#CMT2022; LaboPass) and the forward and reverse primers with the T7R sequence. The primers used in this study are listed in Supplementary Table S1. Each tube containing a mixture of the PCR product, 20 U ribonuclease inhibitors (N2111; Promega, WI, United States), 1× DIG RNA labeling mix (#11 277 073 910; Roche, Switzerland), 1× T7 RAN polymerase buffer, 5 mM DTT, and 50 U T7 RNA polymerase (#2540A; Takara Bio Inc., Japan) was incubated for 3 h at 37°C. After freezing with 70% ethanol diluted with RNase-free water and 10 M LiCl for 2 h at −80°C or overnight at −20°C, the tube was centrifuged at 12,000 rpm for 10 min, the supernatant was removed, and the precipitate was dried. After elution with RNase-free water, the tube was treated with DNase I (#04 716 728 001; Roche) for 30 min at 37°C to remove residual DNA. After freezing, centrifuging, and drying, the precipitate was eluted in ISH solution (5× SSC, 50% deionized formamide, 50 μg/mL heparin, 50 μg/mL yeast t-RNA, 1 mM EDTA, 0.1% CHAPS, 2% casein sodium salt, and 20% Tween 20 diluted in DEPC; pH 7.5) at a concentration of 1 μg/mL. Probes were stored at −80°C or −20°C.

Slides for ISH were prepared only by cryosectioning. Before staining, slides were dried for 1 day to prevent the detachment of tissues from the slides. After washing the slides thrice with PBT (0.1% Tween 20, 1× PBS diluted in sterile distilled water) for 5 min each time, they were bleached with H_2_O_2_ solution (#S2023; Dako) for 5 min. The slides were washed and then incubated with 10 μg/mL proteinase K solution (#PF-1048-050-02; Biosesang, Korea) for 1–5 min according to the age of the tissue. These slides were washed again and fixed with 4% PFA for 10 min. Subsequently, they were washed once more and blotted with each pre-warmed probe in ISH solution for 1 day at 68°C. Next day, to stabilize the probes blotted, slides were incubated thrice with pre-warmed high-concentration SSC solution (50% ionized formamide, 6× SSC, and 1% SDS diluted in sterile distilled water) for 15 min at 68°C. Then, they were incubated thrice with pre-warmed low-concentration SSC solution (50% ionized formamide and 2.4× SSC diluted in sterile distilled water) for 15 min at 68°C. After washing the slides with TBST for 5 min at room temperature thrice, they were blocked with 5% inactivated FBS blocking solution for 1 day and then blotted with α-DIG antibody (#11 093 274 910; Roche) overnight at 4°C. Next day, after washing the slides thrice with TBST for 30 min each time, they were incubated thrice with NTMT solution for 10 min. Then, the slides were incubated with the mixture of 250 μg/mL NBT (#11 585 029 001; Roche) and 130 μg/mL BCIP (#11 383 221 001; Roche) for 1–2 days depending on their color. They were then incubated with 1 mM EDTA-diluted PBT for 30 min to stop the colorization reaction and mounted.

All experiments were conducted using at least four biological replicates from independent experiments in both coronal and sagittal directions.

### 2.8 RNA sequencing

After sampling the heads of WT (n = 3) and *Prok2* KO (n = 3) mice at E14.5 with the nasal cavity and brain, including the OB, the samples were divided into each anterior or posterior region by cutting them vertically (in the coronal plane) at the initial level of the putative OB, which was defined based on its first appearance in the rostral-to-caudal series of sections. After tissues were homogenized in TRIzol reagent (#1559618, Invitrogen, Waltham, MA, United States), RNA was extracted via TRIzol and on-column DNase I treatment. cDNA library was prepared using TruSeq Stranded mRNA (Illumina, San Diego, United States) and sequenced using Illumina NovaSeq. Statistics were analyzed based on the fold change (fc), exactTest using edgeR, and hierarchical clustering. P-value was adjusted using Benjamini–Hochberg correction (bh.pval).

## 3 Results

### 3.1 *Prok2* KO mice with OB hypoplasia and olfactory dysfunction were suitable as a mouse model of KS with olfactory disorders

Similar to the hypoplastic OB found in patients with KS (Bortolotto Felippe Trentin et al., 2023; Sarfati et al., 2010; Wakabayashi et al., 2021), the OB of *Prok2* KO mice was smaller than that of WT mice when viewed in the medially dissected head (dashed line in Figure 1A). This OB hypoplasia of *Prok2* KO mice was also shown in the axonal direction (Supplementary Figure S1). Although olfactory dysfunction in patients with KS has been studied (Bortolotto Felippe Trentin et al., 2023; Wakabayashi et al., 2021), the olfactory function of *Prok2* KO mice has not been investigated yet. To determine the ability of *Prok2* KO mice to detect and recognize olfactory signals, olfactory behavior test was conducted on these mice. We observed that the avoidance time against TMT in KO mice was lower than the avoidance time against water in KO mice (Figure 1B). The avoidance time against TMT in KO mice (−28.83 s ± 10.85) was significantly lower than that in WT mice (40.71 s ± 11.82). Thus, these results indicate that *Prok2* KO mice have impaired olfactory cognition.

Olfactory cognition is the unified response of both central and peripheral olfactory systems. To identify whether the disruption of the olfactory cognition in *Prok2* KO mice originated from the peripheral olfactory system, the electrical signal against odor stimuli in KO mice was measured by EOG. According to the results (Figure 1C), the electrical signal against 0.01 M (−11.21 mV ± 3.33) or 1 M (−13.00 mV ± 3.83) amyl acetate in WT mice was significantly higher than that against the vehicle (−2.93 mV ± 0.79). In contrast, the electrical signal against 0.01 M (−4.96 mV ± 1.89) or 1 M (−5.76 mV ± 2.14) amyl acetate in *Prok2* KO mice was not significantly higher than that against the vehicle (−1.92 mV ± 0.78). Therefore, the dose-dependent response against odor stimuli in *Prok2* KO mice reduced. Eventually, the reduced function of the peripheral olfactory system affected the disruption of olfactory cognition in *Prok2* KO mice. Thus, our findings demonstrate that *Prok2* KO mice have olfactory dysfunction similar to patients with KS.

### 3.2 Dysgenesis of olfactory structure in adult *Prok2* KO mice

**FIGURE 2:**
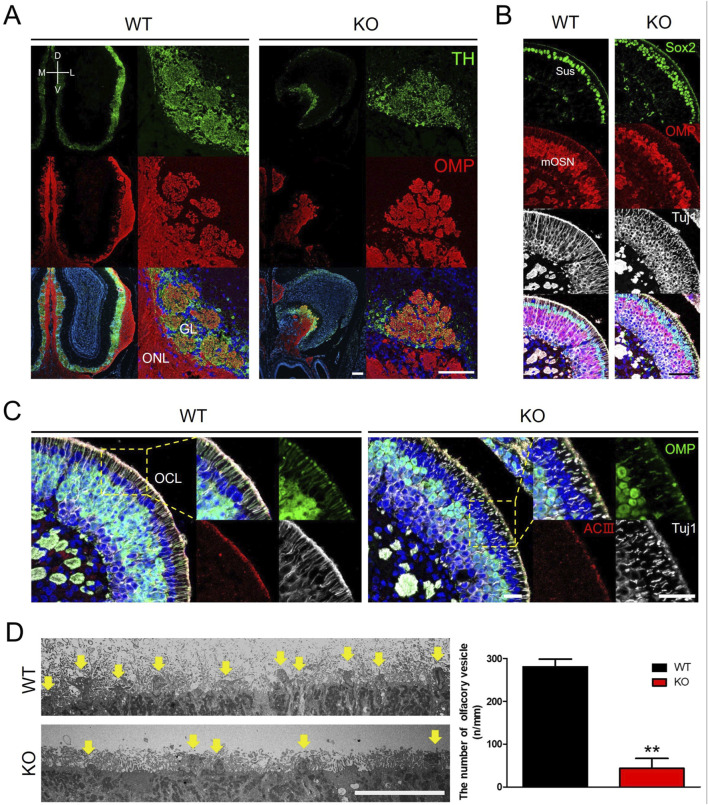
Dysgenesis of the olfactory bulb, olfactory epithelium, and olfactory cilia in adult *Prok2* KO mice Immunostaining images, transmission electron microscopy (TEM) images, and a graph showing the number of olfactory knobs in WT and *Prok2* KO mice at 16 weeks **(A)** Immunostaining of the olfactory bulb with α-TH (green) and α-OMP (red) antibodies. **(B)** Immunostaining of the olfactory epithelium with α-Sox2 (green), α-OMP (red), and α-Tuj1 (white) antibodies. **(C)** Immunostaining of the olfactory epithelium and olfactory cilia with α-OMP (green), α-ACIII (red), and α-Tuj1 (white) antibodies. Yellow dotted rectangles indicate the highly magnified portion (×40) of the images. **(D)** TEM image of the olfactory cilia (yellow arrows) and a graph of the number of olfactory vesicles. Differences between the number of olfactory knobs in WT (n = 3) and *Prok2* KO mice (n = 3) were considered significant at p = 0.0015. Data are presented as mean ± SD. GL, glomerular layer; ONL, olfactory nerve layer; Sus, sustentacular cells; mOSN, mature olfactory sensory neuron; OCL, olfactory cilia layer; D, dorsal; V, ventral; M, medial; L, lateral. Scale bar, 200 μm in **(A)** and **(B)**; 100 μm in **(C)**; and 10 μm in **(D)**. In all panels, n indicates biologically independent repeats. P values were determined using two-tailed unpaired Student’s t-test to compare two groups; *, p < 0.05; **, p < 0.01; ns, non-significant.

Abnormalities in the OB and olfactory function in *Prok2* KO mice implicate dysgenesis in the olfactory system comprising both OB and OE. The radial structure of the glomeruli of the OB was normally formed in WT mice (Figure 2A). In contrast, a few glomeruli with interacting TH^+^ periglomerular neurons and OMP^+^ olfactory axons were formed in KO mice (Figure 2A). In the OE, the Sox2^+^ sustentacular layer was multilayered, OMP^+^ mature OSN layer was thinner, and OMP^−^Tuj1^+^ immature OSN layer in KO mice was thicker compared with that in WT mice (Figure 2B). The result showed that the overall cellular composition of KO mice was disrupted. At a higher magnification, ACIII^+^ or Tuj1^+^ olfactory cilia layer in KO mice was found to be thinner than that in WT mice (Figure 2C). Moreover, the number of olfactory knobs observed via TEM at the higher magnification in KO mice (43.74 n/mm ± 23.67) was significantly less than that in WT mice (280.57 n/mm ± 18.97) (yellow arrows in Figure 2D). Similarly, this relatively reduced the number of olfactory knobs in the OMP^+^ olfactory cilia layer was observed in KO mice (Figure 2C). Therefore, *Prok2* mediated the formation of the glomerular layer (GL) in the OB, the composition of sustentacular cells and OSNs in the OE, the differentiation of immature OSNs into mature OSNs, and the development of the olfactory cilia layer and olfactory knob.

### 3.3 Expression of *Prok2* and its receptor genes in the olfactory system from embryonic development to adulthood

**FIGURE 3:**
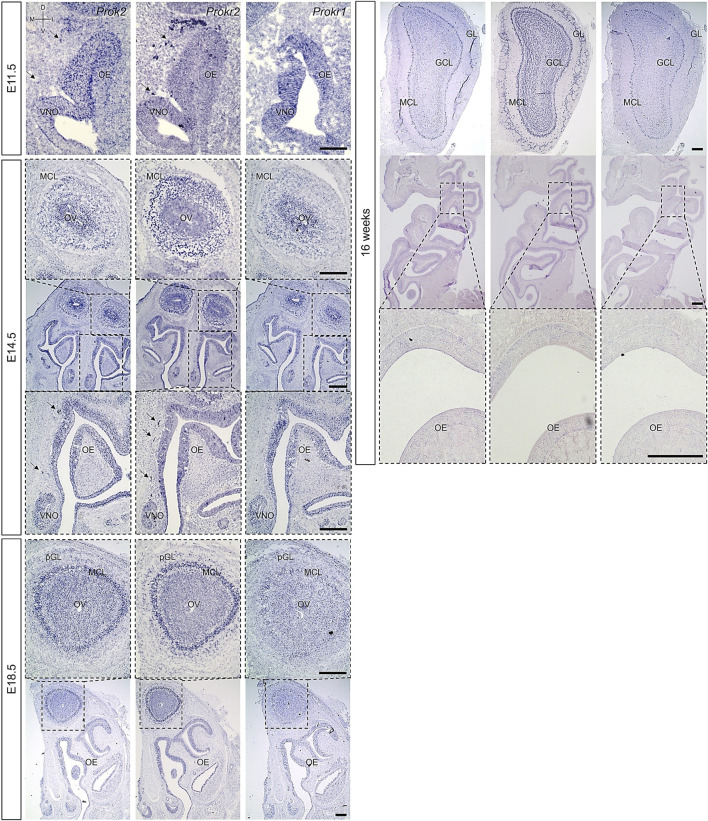
Expression of *Prok2* an its receptors in the olfactory system Images of *in situ* hybridization in the olfactory system with *Prok2*, *Prokr2*, and *Prokr1* probes at E11.5. E14.5, E18.5, and 16 weeks. Black arrows indicate the expression of each gene in the migration route of GnRH neurons, and black dotted rectangles indicate the highly magnified portion (×10, 10×, and 20×) of the images. OE, olfactory epithelium; VNO, vomeronasal organ; MCL, mitral cell layer; OV, olfactory ventricle; (p)GL, (putative) glomerular layer; GCL, granule cell layer; D, dorsal; V, ventral; M, medial; L, lateral. Scale bar, 200 μm.

Previous research has reported that *Prok2* and *Prokr2* are expressed in the migratory route of GnRH neurons from the VNO to the brain at the early embryonic stages, in the olfactory ventricle of putative OB at the embryonic stages, and in most interneurons after birth (Ng et al., 2005; Pitteloud et al., 2007; Wen et al., 2019; Zhang et al., 2007). However, it remains unclear whether *Prokr1*, another receptor gene of *Prok2*, was expressed in olfactory system and whether *Prok2* and its receptor genes were expressed in the OE. To answer these questions, the expression of *Prok2*, *Prokr2*, and *Prokr1* were surveyed at E11.5, E14.5, E18.5, and 16 weeks. *Prokr1* was expressed in the OE and OB during embryonic development and in the OB alone at 16 weeks (Figure 3). This expression pattern is similar to that of *Prok2*. Nevertheless, *Prokr1* was not expressed in the migratory route of GnRH neurons, whereas *Prok2* and *Prokr2* were expressed (arrows in Figure 3). The result indicated that Prok2/Prokr1 signaling is applied in the olfactory system, similar to Prok2/Prokr2 signaling. Besides, *Prok2*, *Prokr2*, and *Prokr1* were all expressed in the OE during embryonic development. In contrast, these genes were not expressed in the OE at 16 weeks (Figure 3). These findings suggest that both Prok2/Prokr2 signaling and Prok2/Prokr1 signaling interacted in the OE and that only the former mediated the migration of GnRH neurons.

In the OB, *Prok2* was expressed in both the olfactory ventricle and other cells, including the mitral cell layer (MCL) at E14.5; in the olfactory ventricle, MCL, and putative GL at E18.5; and in the MCL, part of the GL, and part of the granule cell layer at 16 weeks (Figure 3). Although the expression of *Prokr1* was similar to that of *Prok2*, it was weaker at E18.5 and at 16 weeks (Figure 3). Notably, unlike *Prok2*, *Prokr2* was expressed at a higher level in other cells, including the MCL around the olfactory ventricle at E14.5, than in the olfactory ventricle; it was also broadly expressed in the GL and granule cell layer at 16 weeks (Figure 3). This result is consistent with those of previous studies wherein *Prok2* was expressed at relatively higher levels in mature neurons and *Prokr2* was expressed at relatively higher levels in immature neurons (Ng et al., 2005; Wen et al., 2019). Furthermore, the results indicated that both Prok2/Prokr2 signaling and Prok2/Prokr1 signaling played a role even in the OB.

### 3.4 Disrupted formation of the olfactory structure in *Prok2* KO mice during embryonic development

**FIGURE 4:**
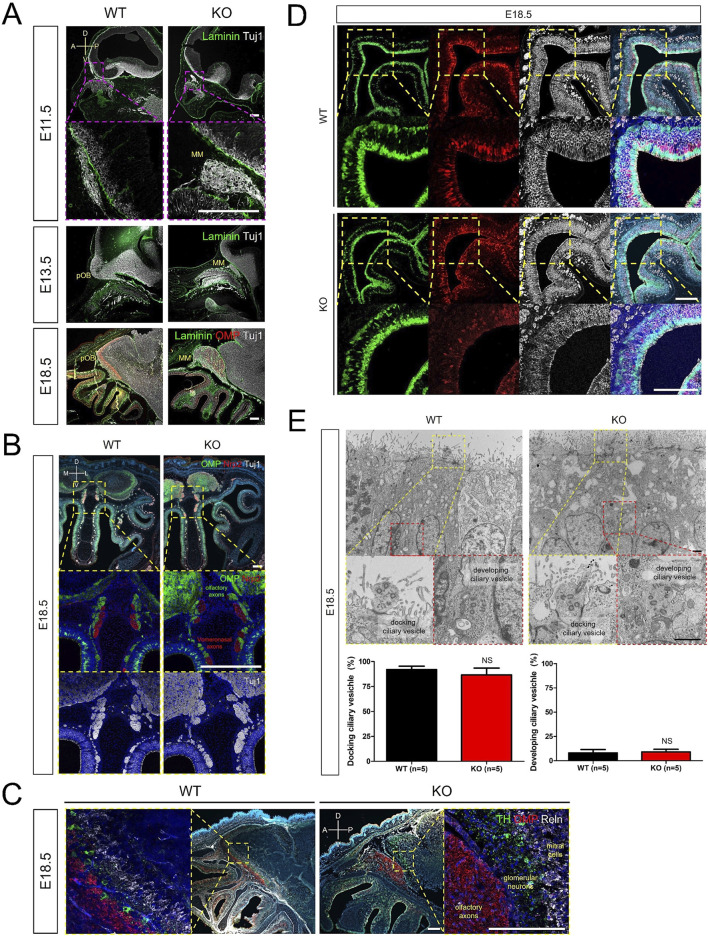
Disruption of olfactory development in *Prok2* KO mouse embryo Images of immunostaining and transmission electron microscopy (TEM) from WT and *Prok2* KO mice at E11.5, E14.5, and E18.5. **(A)** Immunostaining in the forebrain or pOB with α-Laminin (green), α-OMP (red), and α-Tuj1 (white) antibodies. Dotted magenta rectangles indicate the highly magnified portion (×20) of the images. **(B)** Immunostaining in the olfactory nerves and vomeronasal nerves with α-OMP (green), α-Nrp2 (red), and α-Tuj1 (white) antibodies. Dotted yellow rectangles indicate the highly magnified portion (×20) of the images. **(C)** Immunostaining in the pOB with α-TH (green), α-OMP (red), and α-Reln (white) antibodies. Dotted yellow rectangles indicate the highly magnified portion (×20) of the images. **(D)** Immunostaining in the olfactory epithelium (OE) with α-Sox2 (green), α-OMP (red), and α-Tuj1 (white) antibodies. Dotted yellow rectangles indicate the highly magnified portion (×20) of the images. **(E)** TEM image of olfactory vesicles and a graph of the number of olfactory vesicles. The difference in the number of docking olfactory vesicles between WT (n = 5) and *Prok2* KO mice (n = 5) was significant at p = 0.2996, and that in the number of developing olfactory vesicles between WT (n = 5) and *Prok2* KO mice (n = 5) was significant at p = 0.2904. Each dotted rectangle indicates the highly magnified portion (36k ×) of the images. Dotted yellow rectangles represent the docking ciliary vesicles, and dotted red rectangles indicate the developing ciliary vesicles. Data are presented as mean ± SD. MM, migratory mass; pOB, putative olfactory bulb; D, dorsal; V, ventral; A, anterior; P, posterior; M, medial; L, lateral. Scale bar of **(A–D)**, 200 μm; scale bar of **(E)**, 1 μm. In all panels, n indicates biologically independent repeats. P values were determined using the two-tailed unpaired Student’s t-test for comparisons of two groups; ns, non-significant.

Dysgenesis of the olfactory structure in adult *Prok2* KO mice (Figure 2) and the expression of *Prok2* and its receptor genes (Figure 3) implied that Prok2 plays a crucial role in the organization of olfactory structure during embryonic development. Accordingly, the histological changes in *Prok2* KO mice were analyzed during embryonic development. During OB development in WT mice, Tuj1^+^ axons projected and penetrated the Laminin^+^ brain membrane at E11.5, the putative OB with Tuj1^+^ axons increased in size at E14.5, and Tuj1^+^ olfactory axons interacting and radially surrounding the OB expressed OMP, which is a marker for differentiation to mature OSNs, at E18.5 (Figure 4A). In contrast, Tuj1^+^ axons stalled in front of the Laminin^+^ brain membrane in KO mice were observed from E11.5 and Tuj1^+^ and/or OMP^+^ migratory mass in which olfactory axons accumulated increased in size depending on the developmental stages (Figure 4A). Despite the abnormal formation of the putative OB in KO mice, OMP^+^ olfactory axons were discriminated from Nrp2^+^ vomeronasal axons in Tuj1^+^ axons in the migratory route in KO mice at E18.5 (Figure 4B). This discrimination in KO mice was also observed in WT mice (Figure 4B). The results indicated that the GL in the OB was not constituted normally during embryonic development without *Prok2* and that homotypic fasciculation and maturation of olfactory axons in the axon bundles proceeded normally regardless of OB dysgenesis. In addition, OMP^+^ olfactory axons were excluded from TH^+^ glomerular neurons and Reelin + mitral cells in the putative OB of KO mice, whereas most of these axons interacted with each other in the putative OB of WT mice (Figure 4C). Regarding the abnormal formation of GL in KO, few glomeruli in which OMP^+^ olfactory axons and TH^+^ glomerular neurons interacted with each other were constructed at 16 weeks, whereas OMP^+^ olfactory axons were excluded from the putative OB surrounding TH^+^ glomerular neurons at E18.5 (Supplementary Figure S2). This finding indicated that few glomeruli in adult *Prok2* KO were formed after birth.

Sox2^+^ sustentacular cells, Sox2^+^ basal cells, and Tuj1^+^OMP^−^ immature OSNs in the OE of KO mice were similar to those in WT mice (Figure 4D). However, mature OSNs in KO mice were less than those in WT mice (Figure 4D). As depicted by Supplementary Figure S3, *Omp* expression was low at E16.5. In the olfactory knobs of WT mice, both docking ciliary vesicles (95.18% ± 2.01) which arise from the OE surface and developing ciliary vesicles (4.82% ± 2.01) located around the nuclei of sustentacular cells were observed at E18.5 (Figure 4E). The distribution of both docking ciliary vesicles (88.02% ± 5.65) and developing ciliary vesicles (8.41% ± 2.38) in KO mice was not significantly different from that in WT mice (Figure 4E). Prok2 adjusted only the differentiation to mature OSNs in the OE during embryonic development. Consequently, we inferred that Prok2 plays an important role in the organization of the olfactory structure during embryonic development.

### 3.5 Gene set including intermediate filament genes and genes expressed in the putative OB regulated by *Prok2* during olfactory development

**FIGURE 5:**
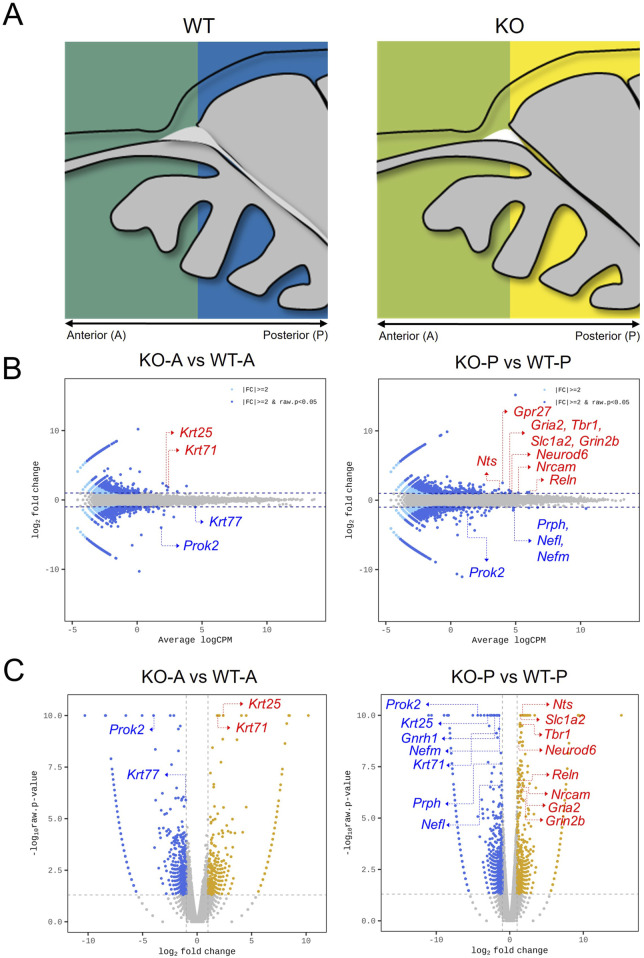
Differentially expressed genes in *Prok2*-deficient olfactory system at E14.5. Transcriptome sequencing of *Prok2* KO mice. **(A)** The anterior and posterior regions of the olfactory tissue. pOB, putative olfactory bulb; OE, olfactory epithelium; D, dorsal; V, ventral; A, anterior; P, posterior. **(B)** Minus-average plot comparing KO (n = 3) and WT (n = 3) mice at each region. Each blue dot and blue arrow indicate the name of each gene. **(C)** Volcano plot comparing KO (n = 3) and WT (n = 3) mice at each region. Each blue dot and blue arrow indicate the name of each gene.

According to the results, fewer OMP^+^ mature OSNs in the OE of *Prok2* KO mice before birth (Figure 4D) led to a reduced proportion of mature OSNs in adults (Figure 2B). Subsequently, reduced response against odor stimuli was observed in the OE (Figure 1C). To figure out what induced the decrease in differentiation to mature OSNs in the OE during embryonic development, the differential expression of genes under *Prok2* deficiency was analyzed at E14.5 when *Omp* was first expressed in the OE. The olfactory tissue of WT or KO mice was divided into anterior or posterior regions. The anterior region included only the OE and the posterior region included both OE and putative OB and the other parts of the brain (Figure 5A). Therefore, the former provided information associated with only the OE and the latter provided information related to the sum of OE, OB, and the other parts of the brain. By sequencing the whole RNA genome in each region, genes with high expression level (higher value of average logCPM in each gene than that in *Prok2*) and significantly greater difference between WT and KO mice (|fc| > 2.0, and bh.pval <0.001) were analyzed. According to the result, keratin genes (*Krt77*, *Krt71*, *Krt25*) in the anterior region displayed significant differences between KO and WT mice (bh.pval = 7.43E-04, 7.25E-11, and 6.58E-13 for *Krt77*, *Krt71*, and *Krt25*, respectively, in Figures 5B,C). In the posterior region, intermediate filament genes (*Prph*, *Nefl*, *Nefm*), ion channel genes for glutamate (*Gria2*, *Grin2b*, *Slc1a2*), mitral-cell-marker genes (*Tbr1*, *Reln*), and other neuronal genes (*Nrcam*, *Nts*, *Neurod6*) had significant differences between KO and WT mice (bh.pval = 8.60E-06, 6.91.E−05, 7.31E-09, 1.02E-04, 2.38E-04, 9.31E-09, 1.75E-07, 4.32E-05, 8.22E-05, 7.25E-10, and 1.01E-06 for *Prph*, *Nefl*, *Nefm*, *Gria2*, *Grin2b*, *Slc1a2*, *Tbr1*, *Reln*, *Nrcam*, *Nts*, and *Neurod6*, respectively, in Figures 5B,C). *Krt77*, *Prph*, *Nefl*, and *Nefm* were downregulated in KO mice, whereas the remaining genes were upregulated compared to those in WT mice (Figures 5B,C).

Similarly, the differentially expressed genes among *Prok2*-relevant genes, KS-relevant genes, OB-constituent genes, OE-constituent genes, and olfactory receptor genes were also analyzed. According to the results, *Gnrh1* was significantly expressed at lower levels in KO mice than in WT mice at the posterior region, as expected (bh.pval = 3.32E-11 in Supplementary Table S3). *Nrcam* and *Arx* were significantly expressed among the genes at the posterior region (bh.pval = 8.22E-05 and 3.47E-04 for *Nrcam* and *Arx*, respectively, in Supplementary Table S3). However, OE-constituent genes were not significantly expressed at all regions between KO and WT mice (higher value of average logCPM in each gene than in *Prok2*, |fc| > 2.0, and bh.pval <0.001 in Supplementary Table S3). Although several genes for olfactory receptors have a higher value of fold change in KO mice than in WT mice, their expression was substantially lower than that of other genes (average logCPM < −2.0 in Supplementary Table S3). For this reason, it was not obvious that *Prok2* affected the differential expression of both OE-constituent genes and olfactory receptor genes.

The heatmap of the genes expressed significantly at each region is described in Supplementary Figure S4. Differentially expressed genes were classified into 6 clusters without *Prok2*. Cluster 1 included genes for glutamate receptor (*Gria2*, *Grin2b*) and glutamate transporter (*Slc1a2*), genes expressed in mitral cells or interneurons in the putative OB (*Tbr1*, *Reln*, *Neurog2*, *Neurod6*, *Arx*), and adhesion molecule gene (*Nrcam*). Clusters 2 and 3 included intermediate filament genes (*Prph*, *Nefm*, *Nefl*) with *Gnrh1*. Cluster 4 included neuropeptide genes (*Nts* and *Npy*). Lastly, clusters 5 and 6 included keratin genes (*Krt71*, *Krt25*, *Krt77*). Thus, the results indicate that *Prok2* mediated the development of the entire olfactory system by regulating the expression of several downstream genes at E14.5.

### 3.6 Downstream genes of *Prok2* during the embryonic development modulated OB formation and affected differentiation into mature OSNs in the OE at later stages

To explore the role of *Prok2*-regulated downstream genes in organizing the olfactory structure during embryonic development, we examined the expression of downstream genes in the olfactory structure across different developmental stages. Initially, we investigated where the genes noted in Supplementary Figure S4 were expressed in the olfactory system of WT mice at E14.5 (Supplementary Figure S5). According to previous reports on *Reln* and *Npy* expression (Ragancokova et al., 2014; Rich et al., 2018), *Reln* was expressed in the MCL, *Npy* was expressed in both MCL and olfactory nerve layer (ONL), and *Nts* was expressed in the MCL similar to *Reln* (thick arrows and dashed line arrows in Supplementary Figure S5). *Slc1a2*, *Gria2*, and *Grin2b* were expressed in the brain with the putative OB (Supplementary Figure S5). *Nefm* and *Nefl* were expressed in both OE and putative OB, as well as in the migratory route of GnRH neurons (black arrows in Supplementary Figure S5). *Nrcam* was expressed in the NFJ and OB similar to *Nefm* and *Nefl*, but not in the OE (black arrows in Supplementary Figure S5). *Krt77* was expressed in the OE, putative OB, epidermis, and whisker follicles, whereas *Krt25* was expressed only in the epidermis and whisker follicles (Supplementary Figure S5). Additionally, *Neurod6* and *Krt77* were expressed in both OE and putative OB (Supplementary Figure S5). Based on the expression of each gene at E14.5, the multi-gene expression in the specific regions of the olfactory structure was explored at E11.5, E14.5, E18.5, and 16 weeks.

**FIGURE 6:**
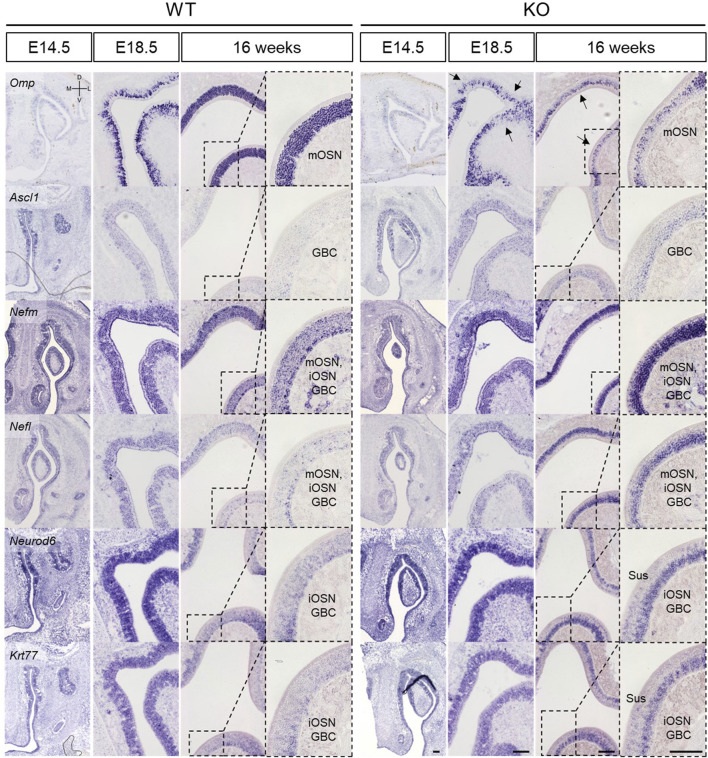
Abnormal development of the olfactory epithelium in *Prok2* KO mice at E14.5, E18.5, and 16 weeks *In situ* hybridization images of the olfactory epithelium with probes for *Omp*, *Ascl1*, *Nefm*, *Nefl*, *Neurod6*, and *Krt77* at E14.5, E18.5, and 16 weeks. Dotted black rectangles indicate the highly magnified portion (×20) of the images. Black arrows in *Prok2* KO mice at E14.5 and E18.5 indicate *Omp* expression. mOSN, mature olfactory sensory neuron; iOSN, immature olfactory sensory neuron; GBC, globose basal cell; Sus, sustentacular cells. Scale bar, 200 μm.

In the OE, *Nefm*, *Nefl*, *Neurod6*, and *Krt77* were analyzed at E11.5, E14.5, E18.5, and 16 weeks together with *Omp* and *Ascl*. It was found that *Omp* expression became more dependent on the developmental stage in WT mice (Figure 6). However, *Omp* expression in KO mice was less than that in WT mice at E18.5 (black arrows in Figure 6). Nevertheless, other genes, including *Ascl1*, which is a marker of globose basal cells (GBCs), were expressed in KO mice similar to those in WT mice at E14.5 and E18.5 (Figure 6). This result suggested that *Nefm*, *Nefl*, *Neurod6*, and *Krt77* expression did not affect the suppression in *Omp* expression during embryonic development. In contrast, considering the locations of *Omp* and *Ascl1* (Figure 6) and the cellular composition mentioned above (Figure 2B) in KO mice at 16 weeks, *Nefm* and *Nefl* were expressed in mature OSNs, immature OSNs, and basal cells, whereas *Neurod6* and *Krt77* were expressed in both sustentacular cells and GBCs at 16 weeks (Figure 6). The findings indicate that cellular composition was disrupted after birth.

**FIGURE 7:**
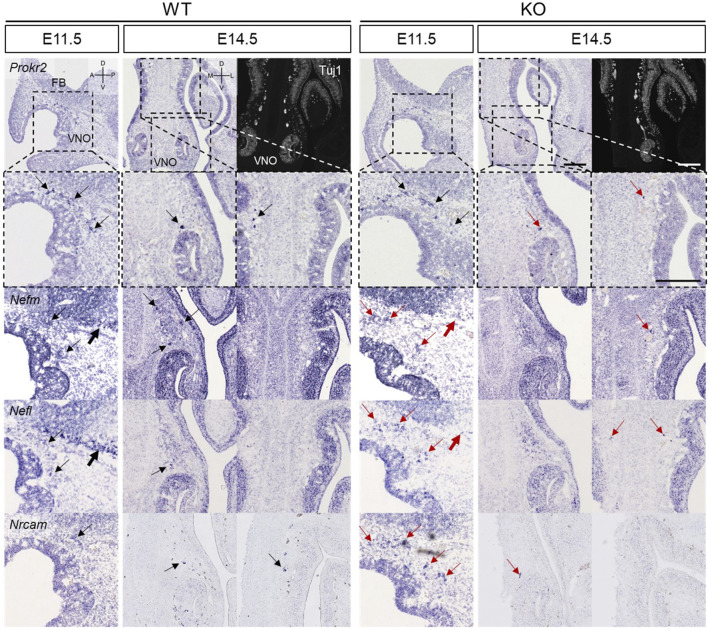
Abnormal development of the VFJ in *Prok2* KO mouse embryos at E11.5 and E14.5 *In situ* hybridization images of the olfactory epithelium with probes of *Prokr2*, *Nefm*, *Nefl*, and *Nrcam* at E14.5, E18.5, and 16 weeks and IF images stained with α-Tuj1 antibody. Dotted black rectangles indicate the highly magnified portion (×20) of the images. Thin arrows indicate the expression of each gene in the migration route of GnRH neurons, and thick arrows indicate the basal region of the brain. Black arrows represent the normal gene expression, and red arrows indicate the difference in gene expression between WT and *Prok2* KO mice. FB, forebrain; VNO, vomeronasal organ; D, dorsal; V, ventral; A, anterior; P, posterior; M, medial; L, lateral. Scale bar, 200 μm.

In the NFJ, *Nefm*, *Nefl*, and *Nrcam* expression were analyzed at E11.5 and E14.5 together with *Prokr2*. *Prokr2* was expressed near the forebrain at E11.5 in both WT and KO mice (Figure 7). However, the spots that expressed *Nefm*, *Nefl*, and *Nrcam* in the NFJ of WT mice appeared cohesive in KO mice (thin arrows in Figure 7). Furthermore, the linear arrangement of the spots that expressed *Nefm* and *Nefl* in the basal region of the forebrain in WT mice was absent in KO mice (thick arrows in Figure 7). At E14.5, the *Nefm* ^+^ *Nefl* ^+^ *Nrcam* ^+^ spots which appeared in the NFJ of WT mice appeared less cohesive in KO mice (thick arrows in Figure 7). Previous studies have reported that GnRH neurons migrate from the VNO, penetrate the membrane of the brain, and bypass the basal region of the brain (Forni and Wray, 2015; Schwanzel-Fukuda et al., 1994; Taroc et al., 2020a; Taroc et al., 2017). Consistent with these findings, our results showed that migration was halted and the migratory route at the basal region of brain was disrupted in KO mice at E11.5. Moreover, it indicated that migration was reduced in KO mice at E14.5.

**FIGURE 8:**
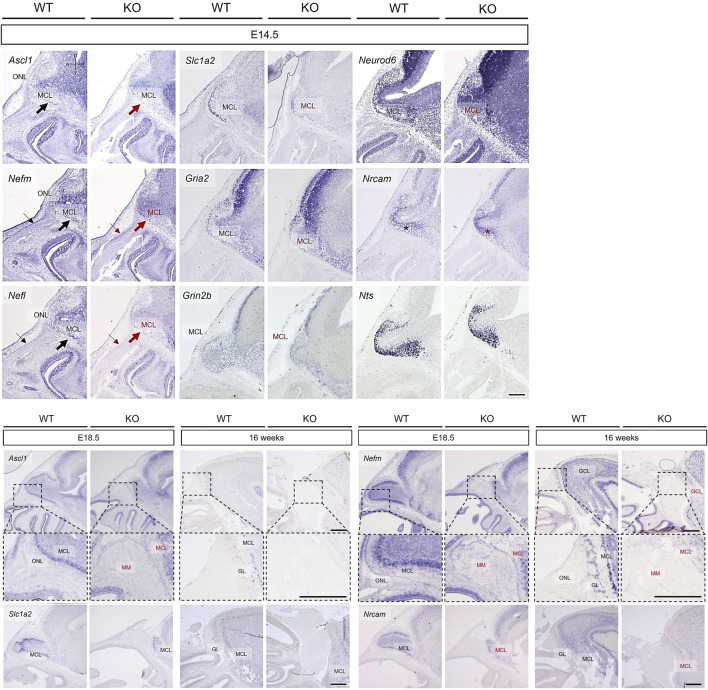
Abnormal development in the olfactory bulb of *Prok2* KO mice at E14.5, E18.5, and 16 weeks. *In situ* hybridization images of the olfactory bulb stained with probes for *Ascl1*, *Nefm*, *Nefl*, *Slc1a2*, *Gria2*, *Grin2b*, *Neurod6*, *Nrcam*, and *Nts* at E14.5, E18.5, and 16 weeks. Thin arrows indicate the expression of each gene in the inner epidermis following the dorsal line of the rostrum in the WT mice, but not in KO mice. Thick arrows indicate the basal region of the brain. Black arrows and characters represent the normal gene expression, while red arrows and characters indicate the difference in gene expression between WT and *Prok2* KO mice. Dotted black rectangles indicate the highly magnified portion (×20) of the images. ONL, olfactory nerve layer; MCL, mitral cell layer; MM, migratory mass; GL, glomerular layer; GCL, granule cell layer. Scale bar, 200 μm.

In the OB, the expression of *Nefm*, *Nefl*, *Nrcam*, *Neurod6*, *Slc1a2*, *Gria2*, *Grin2b*, and *Nts* were analyzed at E14.5, E18.5, and 16 weeks with that of *Ascl1*. Based on the *Ascl1* expression, the MCL and ONL in the putative OB were normally developed in WT mice (Figure 8). Although the putative OB protruded similar to that in WT mice, the MCL and ONL were absent in KO mice (Figure 8). The abnormal development of the MCL in KO mice were also indicated by the expression of *Nefm*, *Nefl*, *Slc1a2*, *Gria2*, and *Neurod6* (Figure 8). Parallel to the absent expression of the basal region of the forebrain at E11.5 (thick arrows in Figure 7), *Nefm* and *Nefl* were not notably expressed in the basal region of the putative OB (thick arrows in Figure 8). Additionally, these filament genes were expressed in the inner epidermis following the dorsal line of the rostrum in WT mice but not in KO mice (thin arrows in Figure 8). Furthermore, neuronal stem cells were cohesive in KO mice, whereas they were scattered in WT mice consistent with the expression of *Nrcam* (asterisks in Figure 8). The OB increased in size and OB layers were visible at E18.5 and 16 weeks in WT mice (Figure 8). Consistent with the expression of *Ascl1*, *Nefm*, *Nefl*, *Nrcam*, and *Slc1a2*, the protrusion of the putative OB from the subventricular zone was delayed, resulting in a larger migratory mass on this putative OB and abnormal development of the MCL in the putative OB of KO mice at E18.5 (Figure 8). Although all layers in the OB were normally developed, the ONL and GL interacting with OB interneurons were absent in KO mice at 16 weeks (Figure 8). Moreover, the MCL in the OB analog developed poorly and the granular cell layer almost did not develop in KO mice (Figure 8). These results suggested that localization of mitral cells expressing *Nefm*, *Nefl*, *Nrcam*, *Neurod6*, *Slc1a2*, *Gria2*, *Grin2b*, and *Nts* are disrupted in *Prok2* KO mice and that *Nefm* and *Nefl* were involved in structural support, including the basal brain and the inner epidermis following the dorsal line of the rostrum.

## 4 Discussion

**FIGURE 9:**
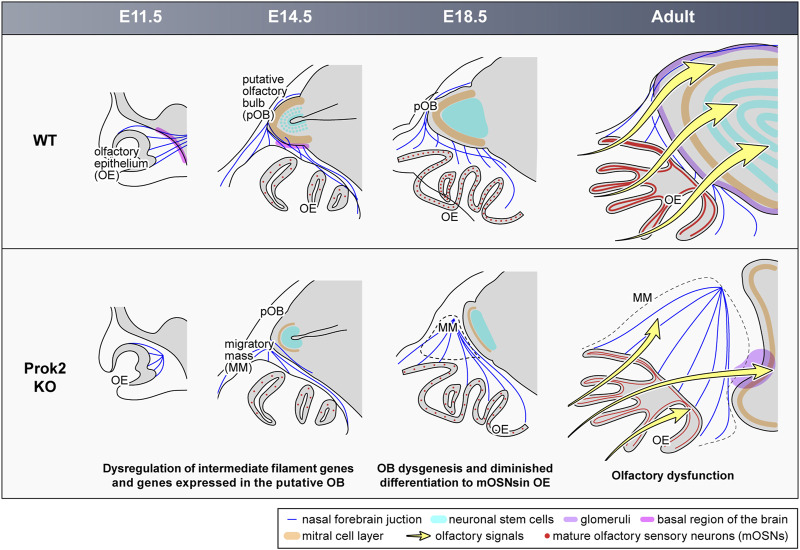
Abnormal development in the olfactory system of *Prok2* KO mice from the embryonic stage to the adult stage. At E11.5, olfactory nerves failed to reach and pass into the basal region of the brain in *Prok2* KO mice. At E14.5, the basal region of the brain and the layers were poorly formed in the putative OB of *Prok2* KO mice. In addition, a migratory mass comprising olfactory nerves adhered to each other appeared. Regardless, the generation of olfactory sensory neurons was normal in the OE of *Prok2* KO mice. At E18.5 and at the adult stage, OB dysgenesis and diminished differentiation to mature OSNs were distinct. This abnormality in the olfactory structure in *Prok2* KO mice was greater at the adult stage than at E18.5. Furthermore, olfactory dysfunction due to the failure to transfer olfactory signals from the OE to the OB were observed in *Prok2* KO mice.

To discover the effects of Prok2 on the olfactory system, the procedures in the formation of olfactory structure were analyzed with respect to the spatiotemporal dynamics and associated molecular events. In summary, intermediate filament genes and genes expressed in putative OB induced structural changes in the olfactory system during embryonic development (Figure 9). Subsequently, fewer mature OSNs in the OE brought about olfactory dysfunction (Figure 9). To the best of our knowledge, the following findings obtained through this research are novel. First, both Prok2/Prokr2 signaling and Prok2/Prokr1 signaling play a role in olfactory development. Second, the interaction between olfactory axons and glomerular neurons or mitral cells in putative OB may contribute substantially to the differentiation into mature OSNs in the OE.

According to previous studies, the main receptor of Prok2 is Prokr2 in the olfactory system (Dodé and Rondard, 2013; Sarfati et al., 2010; Wen et al., 2019). In contrast, our findings showed that Prokr1 was also expressed in the olfactory system similar to Prokr2 but their expression in the migration routes differed. Thus, Prok2/Prokr1 signaling is involved in the olfactory system. Considering the co-expression of Prok2 and Prokr2 in the migration route and the role of Prok2/Prokr2 signaling in the formation of the migration route, unlike Prok2/Prokr2 signaling, Prok2/Prokr1 signaling plays a role in the olfactory system. Future studies in the model limited Prokr2 expression will contribute to a deeper understanding of the precise mechanisms underlying Prok2/Prokr1 signaling in the olfactory system.

Several studies pertaining to the identification of the causes of KS are primarily focused on the migration route of GnRH neurons and subsequent OB formation, as mentioned above (Barraud et al., 2013; Schwanzel-Fukuda et al., 1994; Taroc et al., 2020a; b; Taroc et al., 2017; Watanabe et al., 2009). According to these studies, abnormal development of OB causes congenital anosmia in KS through genes expressed in the NFJ or OB interneurons. However, our results confirmed that olfactory dysfunction in *Prok2* KO mice resulted in the detection of olfactory signals in the OE, that abnormal development of OE occurred as a subsequent event after disrupting the interaction between the olfactory nerve and OB neurons during embryonic development, and that intermediate filament genes and genes expressed in the putative OB were regulated by the *Prok2* gene. These findings demonstrated that olfactory dysfunction in KS affected olfactory signaling in the OE. Furthermore, we speculated that the normal interaction between the olfactory nerves and mitral cells in the putative OB affected the differentiation to mature OSNs.

We found that several intermediate filament genes regulated by *Prok2* gene were expressed in the olfactory system. These genes include keratin genes, neurofilament genes, and *Prph*. Notably, the deficiency of *Prok2* led the suppression of the expression of *Nefm*, *Nefl*, and *Nrcam* together with *Gnrh1* in the NFJ. Considering that the intermediate filaments build the cytoskeletal network by interacting with adhesion molecules (Sanghvi-Shah and Weber, 2017), Prok2 interacts with the migration of GnRH neurons by regulating neurofilaments and neural cell adhesion molecules in the NFJ. Previous studies have reported that axonal fasciculation of homotypic nerves is manipulated by olfactory ensheathing cells and neural cell adhesion molecules in the NFJ in the lamina propria before migrating to the OB (Barraud et al., 2013; Ekberg et al., 2012; Windus et al., 2007). According to these studies, the data on the discrete separation between olfactory axons and vomeronasal axons suggested that Prok2 did not affect the function of olfactory ensheathing cells and neural cell adhesion molecules in the NFJ. In line with this, our data showed that Prok2 did not affect the expression of Sox10, which regulates other genes in olfactory ensheathing cells (Supplementary Table S3). In addition, we observed that Krt25 and Krt77 were expressed in the non-olfactory system similar to the epidermis and whisker follicles, indicating that Prok2 regulates both olfactory region and non-olfactory regions. The disparity in gene expression patterns between Krt25 and Krt77 is evident due to their simultaneous expression in both olfactory and non-olfactory systems.

Moreover, neurofilament genes were expressed even in the OB. Although these genes were suppressed in *Prok2* KO mice, the cluster 1 genes in Supplementary Figure S4 were overexpressed in the OB. This discrepancy in the expression level appears to be influenced by whether the genes were exclusively expressed in the OB. We found that penetration into the membrane of brain and innervation with interneurons, mitral cells, and tufted cells in the OB failed in *Prok2* KO mice. Moreover, the defect in the differentiation of neuronal stem cells to projection neurons or radial glia cells at E14.5, the formation of the MCL and ONL, and subsequent normal development of all OB layers in *Prok2* KO mice failed. It is likely that this abnormal development of OB indicates the overexpression of cluster 1 genes in the OB. Thus, our results demonstrate that Prok2 plays a role in the forebrain surrounding the olfactory nerves and the olfactory nerves penetrating the forebrain.

Despite the abnormal development in the NFJ and OB, no evident defect was found in the OE of *Prok2* KO mice without less differentiation into mature OSNs. Among olfactory receptor genes, some had relatively higher expression levels between KO and WT mice (|fc| > 100, 1.00-E6 < adj.pval <1.00-E2) (Supplementary Table S3). However, these olfactory receptor genes had low expression levels (average LogCPM < −2) in both steady and *Prok2*-deficient states (Supplementary Table S3). Therefore, it cannot be accurately determined whether differentially expressed olfactory receptor genes with *Prok2* cause less differentiation into mature OSNs. Furthermore, it is likely that the indirect signals from other olfactory structures affected the differentiation into mature OSNs, rather than the direct signals in the OE.

Prior studies have reported that tufted cell-like mitral cells encountered olfactory axons and formed fascicles (Blanchart et al., 2006; Ravi et al., 2017; Tufo et al., 2022). Other studies showed that the defasciculating of olfactory axons proceeded and that olfactory axons broke down the brain membrane in the outer ONL for sorting and refasciculating specific glomeruli in the inner ONL (Ekberg et al., 2012; Rich et al., 2018). It implied that many signals were sent and received between the OB and OE during the developmental procedure. Furthermore, regarding our data about the abnormal organization of the MCL and ONL appearing before the differentiation of mature OSNs in the OE of *Prok2* KO mice, it is conferred that the failure to deliver signals from the OB due to poor penetration of olfactory axons into the OB resulted in less differentiation into OSNs in the OE. In particular, olfactory axons did not interact with genes expressed only in the OB, similar to *Nts*, *Gria2*, and *Grin2b*. Considering that glutamate from olfactory axons is used to transfer olfactory signaling into tufted cells expressing *Gria2*, *Grin2b*, or *Slc1a2* in the OB (Gurden et al., 2006; O’Connor and Jacob, 2008), the failure of ONL and MCL formation indicated a low possibility that factors for differentiation to mature OSNs were transferred in the OE. For this reason, these genes likely play a crucial role in the differentiation to mature OSNs in the OE. Thus, our result showed that *Prok2* mediated the differentiation into mature OSNs through the interaction between olfactory axons and the MCL and/or ONL. Unfortunately, more direct evidence is needed to support our hypothesis on the role of *Prok2* in differentiation into mature OSNs. Future studies using direct treatment with neurotransmitters or co-culture of neurons will be required to demonstrate that the genes identified in our study solely affect the differentiation into mature OSNs.

In addition, *Prok2* might affect the cellular composition and development of olfactory cilia in the OE and the formation of GL in the OB, according to the interval between E18.5 and 16 weeks. These organizations are ultimately connected to each other by numerous mature OSNs. Previous studies have reported that olfactory stimulation upon exposure to air after birth facilitated the generation of mature OSNs (Kim et al., 2023; van der Linden et al., 2020). We believe that the differentiation to mature OSNs during the postnatal period was dependent on olfactory stimulation. During both embryonic and postnatal periods in *Prok2* KO mice, differentiation to mature OSNs was reduced. Thus, our data suggested that *Prok2* mediated the differentiation to mature OSNs through both independent and dependent olfactory stimulation. Further research into the differentiation driven by dependent olfactory stimulation is necessary to elucidate the role of *Prok2* in regulating the organization of the olfactory system during the postnatal period.

In summary, we studied the embryonic development of the olfactory system, especially focusing on the OE which has not been adequately addressed in previous studies. The results provide new insight into the development of the olfactory system, indicating that both OB and OE influence normal olfactory function and that their interaction is essential for the development and function of the olfactory system. Moreover, we elucidated the mechanistic role of *Prok2* in the development of the olfactory system through intermediate genes and genes expressed in the putative OB. Finally, this study will aid in identifying the cause of olfactory disorders and advancing developmental or genetic research of the olfactory system.

## Data Availability

The datasets presented in this study can be found in online repositories. The names of the repository/repositories and accession number(s) can be found in the article/Supplementary Material.
