# Supplementary material for: Spatiotemporal regulation by downstream genes of Prok2 in the olfactory system: from development to function
Source: Front Cell Dev Biol. 2025 Jul 22;13:1550845. doi: 10.3389/fcell.2025.1550845 (PMC12321839; doi:10.3389/fcell.2025.1550845)
Supplement: Supplementary file 1 [file DataSheet1.docx]

Supplementary Material

# Supplementary Figures and Tables

## Supplementary Figures

**
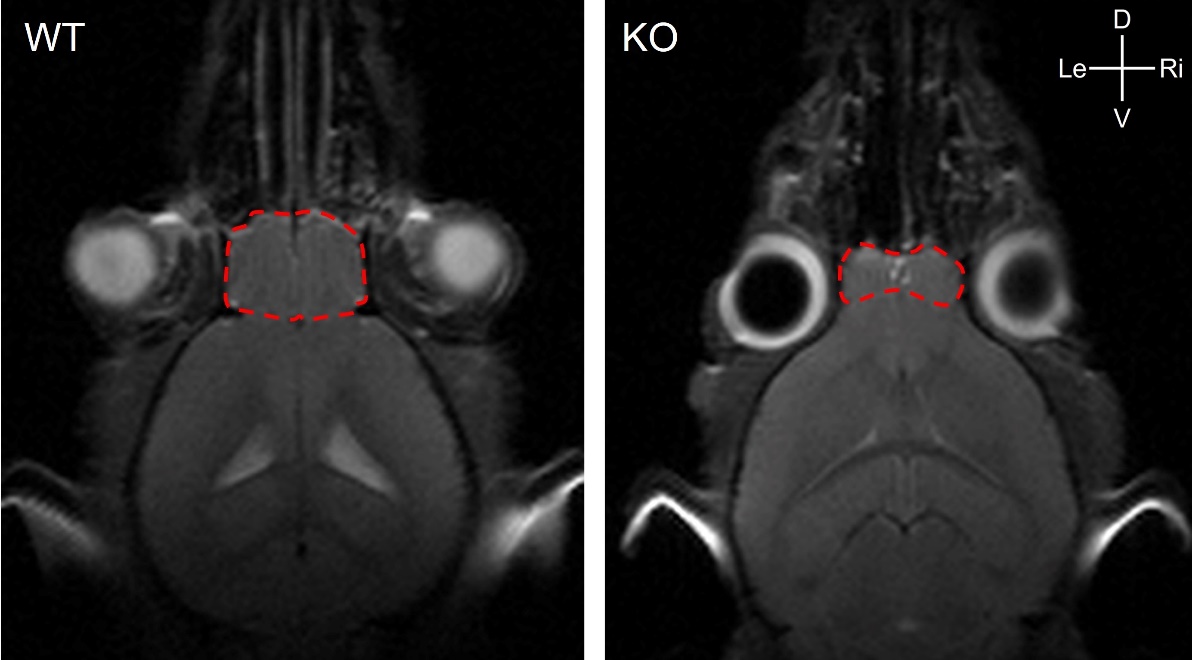
**

**Supplementary Figure 1.** MEMRI images of olfactory bulb (OB) hypoplasia in *Prok2* KO mice at 16 weeks. Each dashed red line represents the OB size. MM, migratory mass; D, dorsal; V, ventral; Le, left; Ri, right.

**
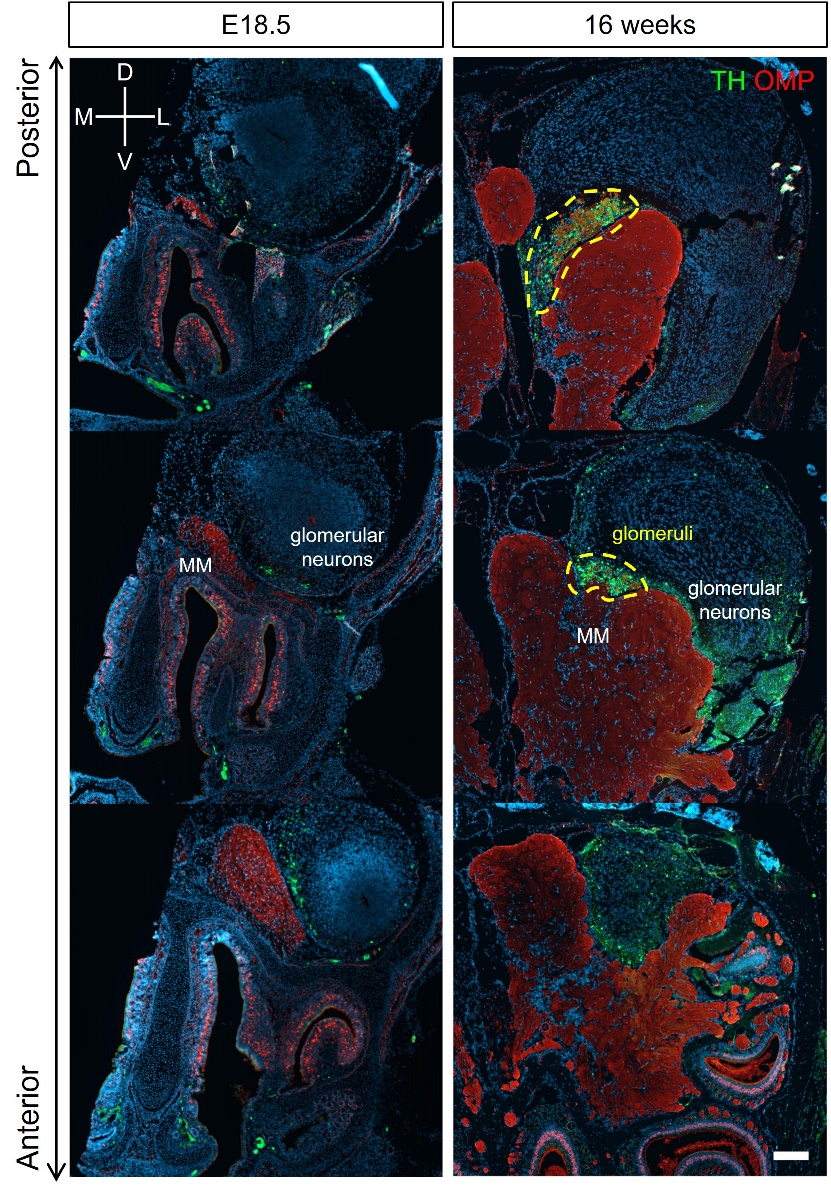
**

**Supplementary Figure 2.** Olfactory bulb (OB) hypoplasia in *Prok2* KO mice at E18.5 vs. 16 weeks. Images showing immunostaining of samples from *Prok2* KO mice with α-TH (green) and α-OMP (red) antibodies at E18.5 and 16 weeks. Dashed yellow lines indicate glomeruli in the OB. MM, migratory mass; D, dorsal; V, ventral; M, medial; L, lateral. Scale bar, 200 μm.


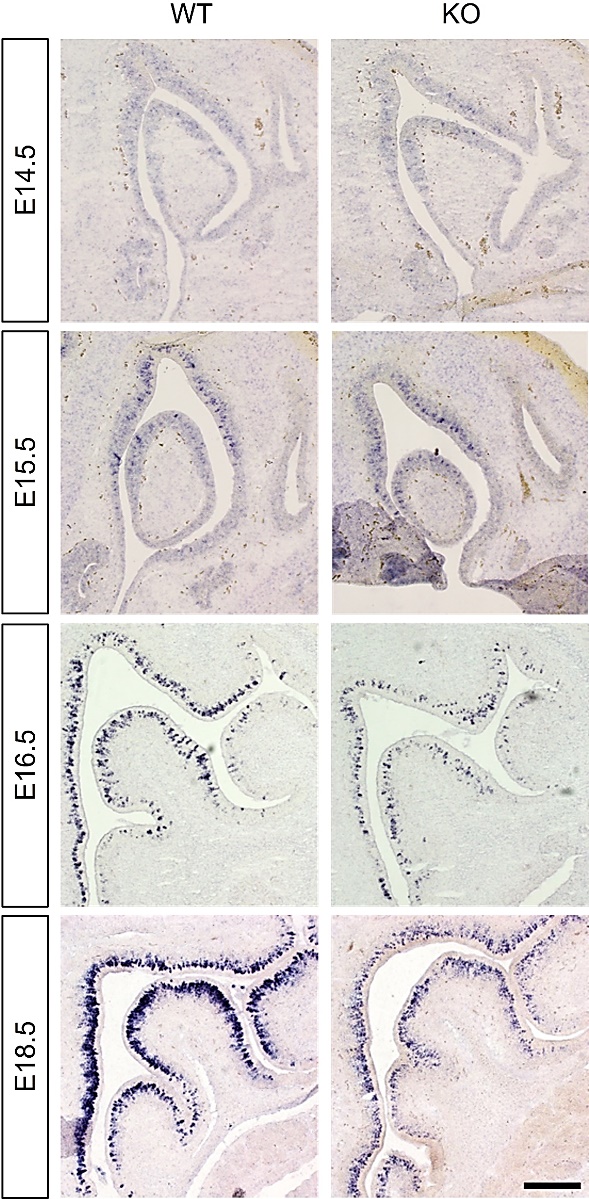


**Supplementary Figure 3.** *Omp* expression in the olfactory epithelium of *Prok2* KO embryos at E14.5, E15.5, E16.5, and E18.5. Images showing *in situ* hybridization with the *Omp* probe in the olfactory epithelium at E14.5, E15.5, E16.5, and E18.5. Scale bar, 200 μm.

**
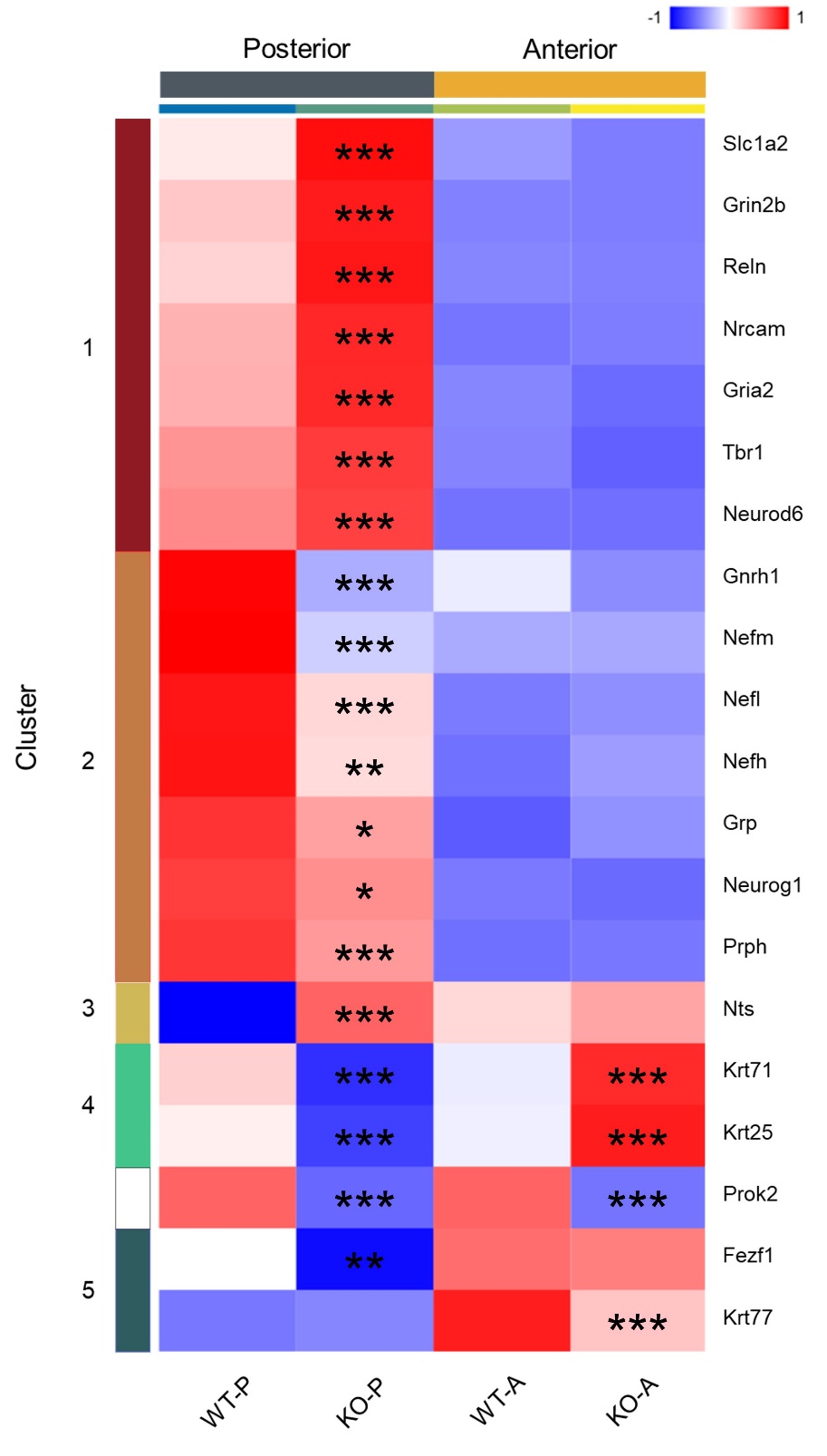
**

**Supplementary Figure 4.** Heatmap comparing KO (n = 3) and WT (n = 3) mice at each region. In all panels, n indicates biologically independent repeats. P values were calculated using exactTest with TMM-normalized counts obtained from edgeR for statistical analysis and hierarchical clustering. The p-value was adjusted using Benjamini–Hochberg correction (bh.pval). Statistical analysis of the comparison between KO and WT mice. *******, bh.pval < 1.00E-15; ******, bh.pval < 1.00E-12; *****, bh.pval < 1.00E-9; ****, bh.pval < 1.00E-6; ***, bh.pval < 1.00E-3.


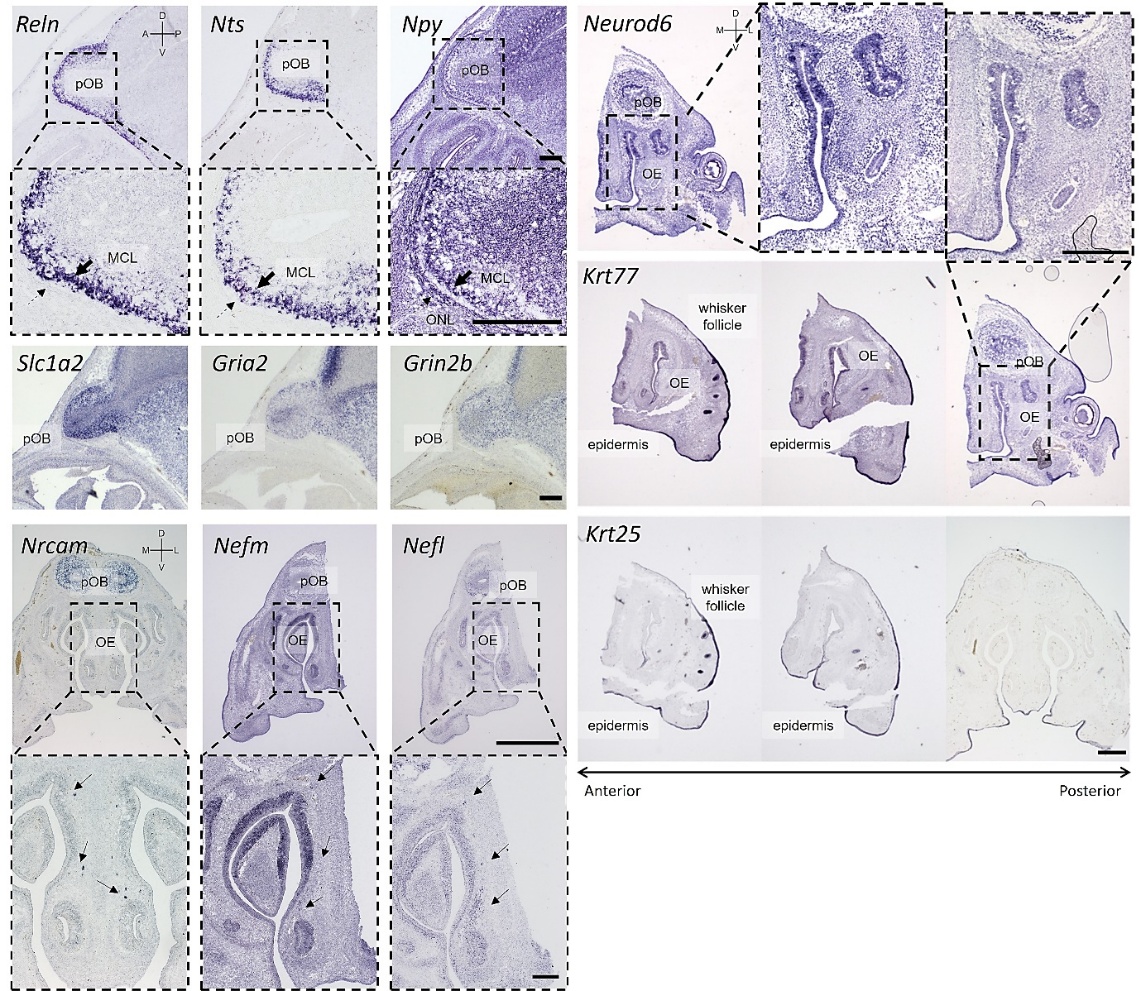


**Supplementary Figure 5.** Expression of *Reln*, *Nts*, *Npy*, *Slc1a2*, *Gria2*, *Grin2b*, *Nrcam*, *Nefl*, *Nefm*, *Krt77*, and *Krt25* in the olfactory structure of WT mouse embryos at E14.5 observed using *in situ* hybridization with probes for the aforementioned genes in the olfactory system at E14.5. Dotted black rectangles indicate the highly magnified portions of the images. Thin dashed black arrows indicate the expression of each gene in the ONL, thick black arrows indicate the expression of each gene in the MCL, and thin black arrows indicate the expression of each gene in the migratory route of hormone neurons. pOB, putative olfactory bulb; MCL, mitral cell layer; ONL, olfactory nerve layer; OE, olfactory epithelium. Scale bar, 200 μm.

## Supplementary Tables

**Supplementary Table 1.** Information about the primers used in this study

| **Purpose** | **Ensemble Accession No.** | **Name** | **Sequence (5′-3′)** |
| --- | --- | --- | --- |
| **Genotyping** | NM_015768.3 | WT-F | CTCCCTTTCACCGTGAAGTTCTCC |
|  |  | WT-R | TAACGAGTAAAACAACAGCCAACAGC |
|  | LT726830.1 | KO-F | GCAGCCTCTGTTCCACATACACTTCA |
|  |  | KO-R | CAAAGGGCAAAAGGAAGTTTCTGC |
| **ISH** | NM_001037539, NM_001170419,  NM_015768 | α-Prok2-F | TGACTCGGAAAAGTCATGTTGC |
|  |  | α-Prok2-T7R | TAATACGACTCACTATAGGGAGAGCTTGGGGGATCTGGTTC |
|  | NM_144944 | α-Prokr2-F | GAGAAGCACTACCTCACCGC |
|  |  | α-Prokr2-T7R | TAATACGACTCACTATAGGGAGATGATACATCTGTGGTAAGCCTG |
|  | NM_001355655, NM_021381 | α-Prok1-F | TATAGGGAGATGATACATCTGTGGTAAGCCTG |
|  |  | α-Prok1-T7R | TAATACGACTCACTATAGGGAGATGCCAAGCTAAGAAGGTGAGT |
|  | NM_011010.2 | α-OMP-F | CGTCTACCGCCTCGATTTCA |
|  |  | α-OMP-T7R | TAATACGACTCACTATAGGGAGAAAGCAGTCAGATCAAGCCCC |
|  | NM_008553.5 | α-Ascl1-F | TCGTCCTCTCCGGAACTGAT |
|  |  | α-Ascl1-T7R | TAATACGACTCACTATAGGGAGACCCATCTGTGATTCGGGCTT |
|  | NM_011261.2 | α-Reln-F | CGCCAAGAACAATACCGCTG |
|  |  | α-Reln-T7R | TAATACGACTCACTATAGGGAGAATCGTGATTCGGTTCCACCC |
|  | NM_024435 | α-Nts-F | GCAAGTCCTCCGTCTTGGAA |
|  |  | α-Nts-T7R | TAATACGACTCACTATAGGGAGAAGGAACCCCTCTTGAGAATGT |
|  | NM_NM_023456.3 | α-Npy-F | CGCCACGATGCTAGGTAACAA |
|  |  | α-Npy-T7R | TAATACGACTCACTATAGGGAGACAGGCAGACTGGTTTCAGGG |
|  | NM_001077514, NM_001077515, NM_001361018, NM_011393 | α-Slc1a2-F | TGCCCAAGCAGGTAGAAGTG |
|  |  | α-Slc1a2-T7R | TAATACGACTCACTATAGGGAGATGATGAGGCCCACGATCAC |
|  | NM_001039195, NM_001083806,  NM_001357924, NM_001357927, NM_013540 | α-Gria2-F | ACAGTGCATTTCGGGTAGGG |
|  |  | α-Gria2-T7R | TAATACGACTCACTATAGGGAGAACTTGAACCTGCTTGAGGGC |
|  | NM_001363750, NM_008171 | α-Grin2b-F | CCTCCTGTGTGAGAGGAAAGAA |
|  |  | α-Grin2b-T7R | TAATACGACTCACTATAGGGAGAAATCGAGGATCTGGGCGATG |
|  | NM_001146031, NM_176930 | α-Nrcam-F | GAAAGCTCAGAGGCCCCTTC |
|  |  | α-Nrcam-T7R | TAATACGACTCACTATAGGGAGATTGCCCACACTGGCTGATAC |
|  | NM_008691 | α-Nefm-F | CGAGAGCAGCCTCGACTTC |
|  |  | α-Nefm-T7R | TAATACGACTCACTATAGGGAGAAATGACGAGCCATTTCCCACT |
|  | NM_010910 | α-Nefl-F | CTCGATCTGAGCCAGGTAGC |
|  |  | α-Nefl-T7R | TAATACGACTCACTATAGGGAGACTGGTGAAACTGAGCCTGGT |
|  | NM_009717 | α-Neurod6-F | TCTGCTCACGTTCGTCCAAA |
|  |  | α-Neurod6-T7R | TAATACGACTCACTATAGGGAGAAGACATTGAAGTATGCTGTGTGC |
|  | NM_001003667 | α-Krt77-F | AGCGGGAACAGATCAAGACC |
|  |  | α-Krt77-T7R | TAATACGACTCACTATAGGGAGACTGCACCGAGATGCTCACTT |
|  | NM_133730 | α-Krt25-F | AGGCATTGGAAGTGGCTTCT |
|  |  | α-Krt25-T7R | TAATACGACTCACTATAGGGAGAACTCCAGAGAGTGTTTCGTGG |

**Supplementary Table 2.** Information about the primary antibodies used in this study

| **Target** | **Company** | **Cat No.** | **Host** | **Poly-/Monoclonal** | **Dilution ratio** |
| --- | --- | --- | --- | --- | --- |
| OMP | Wako | 019-22291 | Goat | Polyclonal | 1:100 |
| OMP | Abcam | ab183947 | Rabbit | Polyclonal | 1:100 |
| TH | Merck | AB152 | Rabbit | Polyclonal | 1:200 |
| Sox2 | Abcam | ab79351 | Rabbit | Polyclonal | 1:50 |
| Tuj1 | Abcam | ab78078 | Mouse | Polyclonal | 1:250 |
| ACⅢ | Santa Cruz | sc-32113 | Goat | Polyclonal | 1:50 |
| Laminin | Sigma-Aldrich | L9393 | Rabbit | Polyclonal | 1:100 |
| Nrp2 | R&D Systems | AF567 | Goat | Polyclonal | 1:50 |
| Reelin | Merck | MAB5364 | Mouse | Monoclonal | 1:50 |

**Supplementary Table 3.** Transcriptional sequencing analysis of *Prok2* KO mice for *Prok2*-relevant, Kallmann syndrome-relevant, OB-constituent, OE-constituent, and olfactory receptor genes. Genes with significantly greater differences between WT and KO mice (|fc| > 2.0 and bh.pval <0.001) and their fc values are indicated in bold characters. The fc value above 0 is shown in red and the value below 0 is shown in blue.

| **Type** | **KO_P/WT_P .logCPM** | **KO_P/WT_P .fc** | **KO_P/WT_P .bh.pval** | **KO_A/WT_A .logCPM** | **KO_A/WT_A .fc** | **KO_A/WT_A .bh.pval** | **Gene** | **Transcript_ID** |
| --- | --- | --- | --- | --- | --- | --- | --- | --- |
| **Prok2-relevant genes** | 1.84 | **-20.44** | **1.00E-38** | 1.85 | **-15.92** | **2.37E-34** | **Prok2** | NM_001037539, NM_001170419, NM_015768 |
|  | -2.49 | **9.56** | **1.74E-02** | -2.64 | -1.35 | 1.00E+00 | **Prok1** | NM_001044382, NM_001357885, XM_017319583 |
|  | 1.83 | 1.55 | 1.89E-01 | 1.73 | -1.02 | 1.00E+00 | Prokr2 | NM_144944, XM_011239555, XM_011239557, XM_011239558 |
|  | 3.93 | 1.20 | 9.73E-01 | 2.76 | 1.05 | 1.00E+00 | Prokr1 | NM_001355655, NM_021381, XM_006506432, XM_006506433 |
|  | 1.32 | **-4.27** | **3.32E-11** | 0.07 | -1.60 | 6.39E-01 | **Gnrh1** | NM_008145, NR_133010, XM_006518564 |
|  | 1.29 | -1.54 | 2.60E-01 | 0.72 | 1.04 | 1.00E+00 | Lhb | NM_008497, XM_006541350, XM_011250810, XM_017322004, XM_030242177 |
|  | -1.87 | -2.24 | 4.61E-01 | -1.52 | 1.21 | 1.00E+00 | Lhcgr | NM_001364898, NM_013582, NR_157357, XM_006523719, XM_006523720, XM_006523721, XM_006523722, XM_006523723, XM_011246310 |
|  | -4.61 | 17.08 | 1.00E+00 | -5.17 | 1.00 | 1.00E+00 | Fshb | NM_008045 |
|  | -4.19 | 33.16 | 7.45E-01 | -5.17 | 1.00 | 1.00E+00 | Fshr | NM_013523 |
|  | 5.20 | **2.11** | **8.22E-05** | 3.06 | 1.04 | 1.00E+00 | **Nrcam** | NM_001146031, NM_176930, XM_006515944, XM_006515945, XM_006515946, XM_006515948, XM_006515949, XM_006515950, XM_006515951, XM_006515952, XM_006515953, XM_006515954, XM_006515955, XM_006515956, XM_006515957, XM_006515958, XM_006515959, XM_006515960, XM_006515961, XM_006515962, XM_006515963, XM_006515964, XM_006515965, XM_006515966, XM_006515967, XM_006515968, XM_017315089, XM_017315090, XM_017315091, XM_017315095, XM_017315096, XM_017315098, XM_017315099, XM_017315100, XM_017315101, XM_017315102, XM_017315103, XM_030246759, XM_030246760, XM_030246761, XM_030246762, XM_030246763, XM_030246764, XM_030246765, XM_030246766, XM_030246767, XM_030246768, XM_030246769, XR_003950054, XR_381525, XR_381526 |
|  | 1.34 | -1.92 | 2.25E-02 | -2.41 | 4.72 | 1.86E-01 | Grp | NM_175012, XM_006525886, XM_006525887 |
|  | 2.19 | -1.82 | 1.92E-02 | -3.00 | -2.41 | 1.00E+00 | Neurog1 | NM_010896 |
|  | 4.23 | 1.04 | 1.00E+00 | 1.26 | -1.01 | 1.00E+00 | Ascl1 | NM_008553 |
| **KS-relevant genes** | 1.84 | **-20.44** | **1.00E-38** | 1.85 | **-15.92** | **2.37E-34** | **Prok2** | NM_001037539, NM_001170419, NM_015768 |
|  | 1.83 | 1.55 | 1.89E-01 | 1.73 | -1.02 | 1.00E+00 | Prokr2 | NM_144944, XM_011239555, XM_011239557, XM_011239558 |
|  | -3.61 | 65.33 | 1.77E-01 | -0.58 | 1.44 | 1.00E+00 | Fgf8 | NM_001166361, NM_001166362, NM_001166363, NM_010205, XM_006526668, XM_011247143, XM_030250752 |
|  | 8.05 | 1.00 | 1.00E+00 | 8.64 | 1.03 | 1.00E+00 | Fgfr1 | NM_001079908, NM_001079909, NM_010206 |
|  | 4.03 | -1.11 | 1.00E+00 | 3.77 | -1.07 | 1.00E+00 | Sox10 | NM_011437 |
|  | 6.73 | 1.25 | 7.45E-01 | 6.86 | 1.03 | 1.00E+00 | Gli3 | NM_008130, XM_006516552, XM_017315389 |
|  | 3.52 | -1.05 | 1.00E+00 | 3.54 | -1.04 | 1.00E+00 | Isl1 | NM_021459 |
|  | 1.29 | **2.41** | **3.47E-04** | -2.85 | 1.68 | 1.00E+00 | **Arx** | NM_001305940, NM_007492 |
|  | 4.19 | -1.77 | 9.54E-03 | 5.11 | -1.05 | 1.00E+00 | Fezf1 | NM_028462, XM_006505175 |
|  | 6.12 | 1.05 | 1.00E+00 | 5.90 | 1.01 | 1.00E+00 | Hs6st1 | NM_015818 |
|  | 6.05 | 1.05 | 1.00E+00 | 6.16 | 1.09 | 1.00E+00 | Wdr11 | NM_172255, XM_030242306, XR_378226 |
| **OB-constituent genes** | 1.84 | **-20.44** | **1.00E-38** | 1.85 | **-15.92** | **2.37E-34** | **Prok2** | NM_001037539, NM_001170419, NM_015768 |
|  | 1.83 | 1.55 | 1.89E-01 | 1.73 | -1.02 | 1.00E+00 | Prokr2 | NM_144944, XM_011239555, XM_011239557, XM_011239558 |
|  | 3.93 | 1.20 | 9.73E-01 | 2.76 | 1.05 | 1.00E+00 | Prokr1 | NM_001355655, NM_021381, XM_006506432, XM_006506433 |
|  | 4.23 | 1.04 | 1.00E+00 | 1.26 | -1.01 | 1.00E+00 | Ascl1 | NM_008553 |
|  | 4.54 | **2.55** | **1.75E-07** | -0.81 | -2.34 | 1.32E-01 | **Tbr1** | NM_009322, XM_006499098 |
|  | 1.88 | **2.48** | **3.46E-05** | -4.36 | 25.21 | 1.00E+00 | **Neurog2** | NM_009718, XM_030252394 |
|  | 1.29 | **2.41** | **3.47E-04** | -2.85 | 1.68 | 1.00E+00 | **Arx** | NM_001305940, NM_007492 |
|  | 6.01 | **2.14** | **4.32E-05** | 4.50 | -1.02 | 1.00E+00 | **Reln** | NM_001310464, NM_011261 |
|  | 3.70 | **3.00** | **7.25E-10** | 3.90 | 1.16 | 1.00E+00 | **Nts** | NM_024435, XM_006513992 |
|  | 4.73 | 1.75 | 1.17E-02 | 1.61 | 1.17 | 1.00E+00 | Sall1 | NM_001371069, NM_001371070, NM_021390 |
|  | 1.72 | **3.52** | **1.57E-09** | 2.90 | -1.16 | 1.00E+00 | **Npy** | NM_023456 |
|  | 0.06 | 1.81 | 1.91E-01 | -2.47 | 1.22 | 1.00E+00 | Ntsr1 | NM_018766 |
|  | 4.64 | **2.42** | **1.01E-06** | -4.58 | -16.86 | 1.00E+00 | **Neurod6** | NM_009717 |
|  | 1.56 | **2.19** | **1.37E-03** | -0.04 | -1.74 | 3.83E-01 | **Gad1** | NM_001312900, NM_008077, XM_006498765, XM_006498766, XM_030247531, XR_001780815, XR_003950660, XR_003950661 |
|  | 0.21 | -1.56 | 4.11E-01 | -2.31 | 1.29 | 1.00E+00 | Th | NM_009377 |
|  | 2.49 | 1.36 | 5.40E-01 | 0.88 | 1.04 | 1.00E+00 | Calb1 | NM_009788 |
|  | 4.92 | -1.22 | 8.97E-01 | 1.02 | -1.42 | 7.46E-01 | Calb2 | NM_001368293, NM_001368294, NM_007586 |
|  | -5.19 | 1.00 | 1.00E+00 | -4.36 | 25.21 | 1.00E+00 | Pvalb | NM_001330686, NM_013645 |
| **OE-constituent genes** | 1.84 | **-20.44** | **1.00E-38** | 1.85 | **-15.92** | **2.37E-34** | **Prok2** | NM_001037539, NM_001170419, NM_015768 |
|  | 1.83 | 1.55 | 1.89E-01 | 1.73 | -1.02 | 1.00E+00 | Prokr2 | NM_144944, XM_011239555, XM_011239557, XM_011239558 |
|  | 3.93 | 1.20 | 9.73E-01 | 2.76 | 1.05 | 1.00E+00 | Prokr1 | NM_001355655, NM_021381, XM_006506432, XM_006506433 |
|  | 3.14 | -1.20 | 9.73E-01 | 0.08 | 1.49 | 7.76E-01 | Omp | NM_011010 |
|  | 5.09 | -1.05 | 1.00E+00 | 5.07 | 1.04 | 1.00E+00 | Adcy3 | NM_001159536, NM_001159537, NM_138305, XM_006514932, XM_006514933, XM_006514934, XM_006514935 |
|  | 4.40 | -1.24 | 8.06E-01 | 3.26 | 1.13 | 1.00E+00 | Gnal | NM_010307, NM_177137, XM_030250337 |
|  | 2.69 | -1.48 | 2.32E-01 | -0.13 | 1.67 | 5.30E-01 | Cnga2 | NM_007724, XM_006527761 |
|  | -0.29 | -2.11 | 7.81E-02 | -2.52 | 2.51 | 9.36E-01 | Cnga4 | NM_001033317, XM_017322183, XM_030242497 |
|  | 6.71 | -1.05 | 1.00E+00 | 6.37 | 1.11 | 1.00E+00 | Gap43 | NM_008083 |
|  | 7.26 | 1.13 | 1.00E+00 | 4.23 | -1.02 | 1.00E+00 | Tubb3 | NM_023279 |
|  | 4.20 | -1.24 | 8.00E-01 | -0.06 | -1.14 | 1.00E+00 | Neurod1 | NM_010894 |
|  | 2.19 | -1.82 | 1.92E-02 | -3.00 | -2.41 | 1.00E+00 | Neurog1 | NM_010896 |
|  | 4.23 | 1.04 | 1.00E+00 | 1.26 | -1.01 | 1.00E+00 | Ascl1 | NM_008553 |
|  | 5.91 | 1.19 | 9.73E-01 | 4.42 | -1.04 | 1.00E+00 | Sox2 | NM_011443 |
|  | 5.12 | -1.02 | 1.00E+00 | -0.15 | -1.35 | 1.00E+00 | Cyp2g1 | NM_013809 |
|  | 5.26 | 1.12 | 1.00E+00 | 5.23 | -1.24 | 1.00E+00 | Hes1 | NM_008235, XM_006521797 |
| **Olfactory receptor genes** | -2.03 | **-255.71** | **1.77E-06** | -3.47 | -7.10 | 6.35E-01 | **Olfr666** | NM_147096 |
|  | -2.07 | **-247.75** | **2.99E-06** | -5.17 | 1.00 | 1.00E+00 | **Olfr77** | NM_146339 |
|  | -2.44 | **-184.07** | **1.73E-04** | -5.17 | 1.00 | 1.00E+00 | **Olfr70** | NM_019485 |
|  | -2.44 | **-184.07** | **1.73E-04** | -5.17 | 1.00 | 1.00E+00 | **Olfr860** | NM_146528 |
|  | -2.87 | **-128.36** | **5.50E-03** | -5.17 | 1.00 | 1.00E+00 | **Olfr701** | NM_001360747, NM_028910, XM_017312235 |
|  | -2.87 | **-128.36** | **5.50E-03** | -5.17 | 1.00 | 1.00E+00 | **Olfr350** | NM_146627 |
|  | -2.87 | **-128.36** | **5.50E-03** | -5.17 | 1.00 | 1.00E+00 | **Olfr1426** | NM_146809 |
|  | -2.95 | **-120.40** | **8.92E-03** | -3.85 | -1.91 | 1.00E+00 | **Olfr1392** | NM_146470 |
|  | -3.02 | **-112.44** | **1.40E-02** | -4.85 | -8.93 | 1.00E+00 | **Olfr569** | NM_147088 |
|  | -3.19 | **-96.52** | **3.46E-02** | -5.17 | 1.00 | 1.00E+00 | **Olfr30** | NM_146878 |
|  | -3.19 | **-96.52** | **3.46E-02** | -5.17 | 1.00 | 1.00E+00 | **Olfr1131** | NM_146658 |
|  | -3.19 | **-96.52** | **3.46E-02** | -5.17 | 1.00 | 1.00E+00 | **Olfr1213** | NM_146898 |
|  | -3.19 | **-96.52** | **3.46E-02** | -5.17 | 1.00 | 1.00E+00 | **Olfr1251** | NM_001011529 |
|  | -0.98 | -1.67 | 6.16E-01 | -2.58 | **162.42** | **2.16E-03** | **Olfr550** | NM_147104, XM_006507878 |
|  | -3.02 | **113.57** | **1.40E-02** | -5.17 | 1.00 | 1.00E+00 | **Olfr180** | NM_001011662 |
|  | -2.87 | **129.65** | **5.50E-03** | -5.17 | 1.00 | 1.00E+00 | **Olfr697** | NM_146599 |
